# *De Novo* donor-specific anti-HLA antibody risk stratification in kidney transplantation using a combination of B cell and T cell molecular mismatch assessment

**DOI:** 10.3389/fimmu.2025.1508796

**Published:** 2025-02-25

**Authors:** Elaine Chou-Wu, Matthias Niemann, Danny Youngs, Idoia Gimferrer

**Affiliations:** ^1^ Immunogenetics/HLA Laboratory, Bloodworks Northwest, Seattle, WA, United States; ^2^ PIRCHE AG, Berlin, Germany

**Keywords:** HLA molecular mismatch, major histocompatibility complex (MHC), risk stratification, kidney transplantation, *de novo* DSA, Snow, PIRCHE-II, HLAMatchmaker

## Abstract

**Introduction:**

The presence of *de novo* donor-specific antibody (dnDSA) has detrimental effect on allograft outcomes in kidney transplantation. As humoral responses in transplantation are elicited targeting non-self-epitopes on donor HLA proteins, assessing HLA mismatches at the molecular level provides a refined means for immunological risk stratification.

**Methods:**

In the present study, we utilized three HLA molecular mismatch assessment algorithms, Snow, HLAMatchmaker, and PIRCHE-II, to evaluate the independent and synergistic association of B cell and T cell epitope mismatches with dnDSA development in a cohort of 843 kidney transplant recipients.

**Results:**

Our results demonstrated that B cell and T cell epitope mismatches at HLA Class I and DRB1/DQB1 loci are remarkably increased in dnDSA-positive recipients, even after normalization by allele mismatch numbers in individual study subjects. Furthermore, elevated Snow, verified eplet mismatches, and PIRCHE-II scores are significantly associated with dnDSA occurrence individually and in combination.

**Conclusion:**

Our findings highlight the value of utilizing B cell and T cell epitope mismatch evaluation in living donor selection and immunological risk stratification to improve transplant outcomes.

## 1 Introduction

The presence of antibodies directed at donor-specific Human Leukocyte Antigen (HLA) (donor-specific antibodies, DSA) is a major cause of antibody-mediated rejection (ABMR) and allograft loss in solid organ transplant recipients. Owing to the advances of HLA antibody identification assays and immunosuppression regimens, current histocompatibility criteria based on the avoidance of unacceptable DSAs prior to transplantation has achieved very favorable short-term kidney transplant outcomes. However, the development of *de novo* DSA (dnDSA) - anti-donor HLA antibodies that emerge after transplantation, still occurs in 13%-30% of kidney transplant recipients, and is strongly associated with chronic allograft dysfunction and inferior graft survival (1).

It is well established that humoral responses in transplantation are elicited against non-self-epitopes, rather than the entire donor HLA proteins. Therefore, evaluating molecular mismatches, i.e., identifying differential HLA epitopes, between transplant donors and recipients provides a more refined measure for immunological risks over the traditional assessment at the antigen level. Several B cell epitope mismatch algorithms have been developed to evaluate the disparity of amino acid compositions, structures, or physiochemical properties of donor HLA proteins. HLAMatchmaker considers eplets, patches of non-self amino acid residues within a 3.0-3.5 Å radius on the structural surface of donor HLA, as potential targets of recipient B cell humoral response (2). Eplets are considered the functional epitopes that determine the specificity and strength of antigen-antibody interaction (3). Previous studies have demonstrated the correlation of eplet mismatches, particularly at HLA Class II DR and DQ loci, with dnDSA development (4) and T cell-mediated rejection (TCMR) in kidney transplant recipients (5). A subset of eplets is designated as “verified eplets” by the HLAMatchmaker program because their predicted antibody reactivities have been confirmed by studies using human and mouse monoclonal antibodies or polyclonal antibodies from parous women and transplant recipients (6).

A recently developed B cell epitope analysis program, Snow, applies amino acid matching based on a combination of two algorithms – Snowflake, a computational pipeline that calculates solvent accessible surfaces area of individual HLA proteins’ amino acids (7), and Snowball, which correspondingly predicts local surface protrusion of amino acids (8), to provide a refined prediction of mismatched B cell epitopes in transplantation. Studies showed that HLA Class I Snowflake scores significantly correlate with the incidence of DSA in kidney transplant recipients and child-specific HLA antibodies during pregnancy (9). Additional B cell epitope mismatch assessment programs include HLA-EMMA, which determines solvent accessible amino acid mismatches on donor HLA molecules (10), and EMS-3D, which compares the differences of surface electrostatic potential between donor and recipient HLA (11).

Alongside the B cell epitope prediction programs, PIRCHE-II (Predicted Indirectly Recognizable HLA Epitopes) is an algorithm that calculates the numbers of donor HLA-derived peptides that can be presented by recipient’s HLA Class II molecules (12). PIRCHE-II scores represent the numbers of potential T cell epitopes involved in indirect CD4 T cell alloreactivity. Studies have shown that elevated PIRCHE-II scores correlate with the occurrence of dnDSA (12, 13), ABMR (14), TCMR (15, 16), and inferior allograft survival (16). As indirect CD4 T cell allorecognition provides pivotal help to B cell proliferation and differentiation in humoral response against the allograft, we theorize that assessing both T and B cell epitope mismatches would provide a more precise immunological risk stratification for transplant recipients than considering T cell and B cell epitopes separately. The goals of the current study are to investigate the independent and synergistic association of verified eplet, Snow, and PIRCHE-II scores with the incidence of *de novo* DSA development in a cohort of kidney transplant recipients, and to compare the correlation of the three molecular mismatch scores.

## 2 Materials and methods

### 2.1 Study subjects

#### 2.1.1 Main cohort

**Figure 1:**
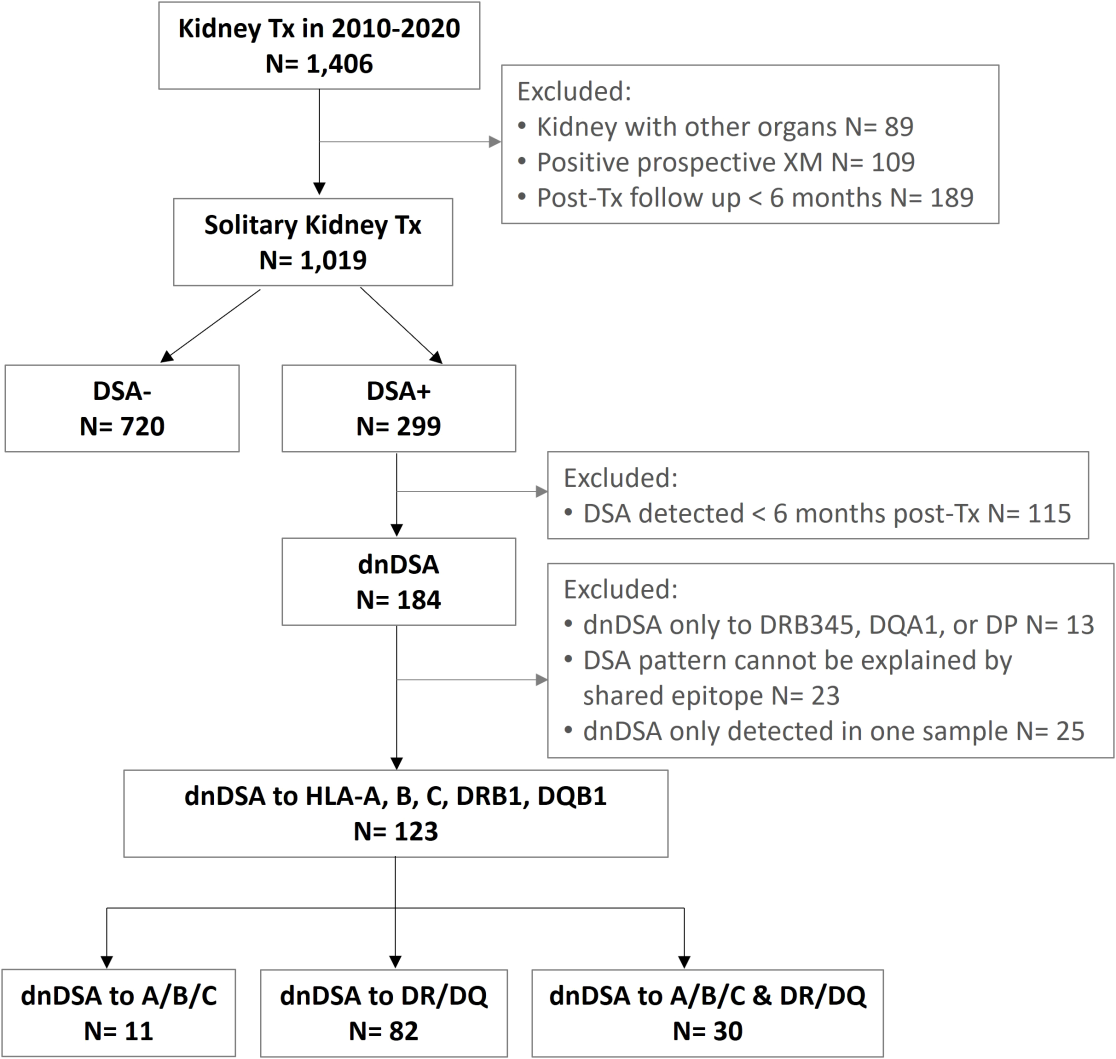
Flow diagram of subject inclusion and exclusion criteria.

The study cohort consists of 736 adult and 107 pediatric patients who underwent a solitary kidney transplant at University of Washington Medical Center, Virginia Mason Medical Centre, Swedish Medical Center, or Seattle Children’s Hospital between 2010 and 2020. All of the patients included in the study were negative for preformed DSAs and had negative flow cytometric crossmatches prior to transplant. *De novo* DSA is defined in this study as DSAs that appeared later than 6 months post-transplant. Patient inclusion and exclusion criteria are depicted in **Figure 1**. This study was reviewed and approved by WCG IRB (study number 1376273).

#### 2.1.2 Validation cohort

To validate the molecular mismatch thresholds for dnDSA risk stratification identified in this study, a validation cohort consists of 544 kidney patients who were transplanted between 2005 and 2010 was constructed using the same inclusion criteria as described above for the main cohort.

### 2.2 HLA genotyping and antibody testing

#### 2.2.1 HLA genotyping and high resolution imputation

HLA-A, B, C, DRB1, and DQB1 low resolution typing of transplant recipients and donors was performed using sequence-specific primers (Micro SSP, One Lambda) or real-time PCR (LinkSeq, One Lambda) following manufacturer-provided protocols. Antigen level typing was used to impute high resolution type using the PIRCHE program. Genotypes with the highest frequency, as determined by haplotype frequencies, were selected as the high resolution type of the study individuals. The ethnicity of the individuals was taken into consideration when available.

#### 2.2.2 Validation of high resolution typing imputation

To verify the validity of using imputed high resolution typing for molecular mismatch assessment, HLA-A, B, C, DRB1, and DQB1 allele-level typing of 285 surrogate transplant pairs performed by Next Generation Sequencing (ScisGo-HLA, Scisco Genetics) was converted to the antigen level and subsequently used for high resolution imputation as described above. Molecular mismatch scores, including Snow, verified eplet, and PIRCHE-II were calculated using the actual and imputed high resolution types.

#### 2.2.3 HLA antibody testing

IgG HLA antibodies were examined in EDTA-treated sera using Luminex-based Single Antigen Bead assay (LABScreen, One Lambda). HLA antibody profiles of DSA-positive patients were thoroughly evaluated based on epitope analysis. Positive DSAs were determined when identifiable shared epitopes are present in at least two consecutive samples using 500 MFI as the cutoff.

### 2.3 HLA epitope mismatch assessment

Epitope mismatches at HLA-A, B, C, DRB1, and DQB1 loci were determined using imputed two field typing after excluding transplant pairs who were allele-matched at the locus/loci of analysis. Snow scores were derived from the Snow algorithm that predicts B cell epitopes based on solvent-accessibility surface area and repeated local protrusion rank (version v4.3, Snow 1.1, IMGT 3.54). Verified eplet mismatch numbers were calculated using HLAMatchmaker program (2) (Class I version 4.0 and Class II version 3.1). Indirect T cell Epitope scores were determined by the PIRCHE-II algorithm considering the Frost binding predictor (version 4.2, Frost 1.1, IMGT 3.54) representing the sum of mismatched donor-derived peptides at HLA-A, B, C, DRB1, and DQB1 loci presented by recipient’s DRB1 proteins. Snow and PIRCHE-II scores are acquired from commercial website http://www.pirche.com; HLAMatchmaker program is publicly downloadable from http://www.epitopes.net/. To confirm the differences of epitope mismatches between groups are independent of allele mismatches, the molecular mismatch scores were divided by the numbers of allele mismatches at the respective loci of analysis for every study subject.

### 2.4 Statistical analysis

Statistical analyses were performed using MedCalc (v20.006) or Prism GraphPad (v9.1.0) software. Normal distribution was determined by D’Agostino & Pearson test. Comparisons of continuous parametric or non-parametric variables were performed with two-tailed unpaired Student t-test or Mann-Whitney test, respectively. Categorical variables were analyzed by Chi-square and Fisher’s exact test. Correlations between molecular mismatch scores were calculated by Spearman’s rank correlation and presented as Spearman’s rank correlation coefficient r_s_. Thresholds of epitope mismatch scores that are associated with dnDSA development were calculated by Receiver Operating Characteristic (ROC) curve, based on the Youden index. dnDSA-free probabilities were examined by Kaplan-Meier curve analysis. Hazard ratios for dnDSA development were determined by univariate and multivariate Cox proportional-hazard regression.

## 3 Results

### 3.1 Patient characteristics

Detailed characteristics of the study subjects are listed in **Table 1**. Among the 843 kidney transplant recipients included in this study, 123 (14.6%) developed dnDSA at one or more of HLA-A, B, C, DRB1, and DQB1 loci. The dnDSA-positive (dnDSA+) group had a significantly higher percentage of pediatric recipients (30.1%), compared to the dnDSA-negative (dnDSA-) subjects (9.7%). Accordingly, dnDSA+ patients had younger median age at transplant (35.7 yo vs. 52.6 yo), younger median donor age (35.8 yo vs. 40.2 yo), a lower percentage of deceased donors (63.4% vs. 73.2%), and a higher proportion of unrelated living donors (24.4% vs. 13.5%), compared to the dnDSA- group. Other recipient characteristics including gender, pre-transplant sensitization, re-transplant, and ABO were comparable between dnDSA-positive and negative groups.

**Table 1:** Demographics of study subjects.

	All	dnDSA -	dnDSA +	p value
	(n= 843)	(n= 720)	(n= 123)	
Recipient				
Recipient sex (male %)	57.1%	56.5%	60.2%	0.452
Recipient age (year), Median (IQR)	50.9 (34.7-62.3)	52.6 (38.2-62.8)	35.7 (15.5-51.2)	<0.0001
Pediatric recipient, n (%)	107 (12.7%)	70 (9.7%)	37 (30.1%)	<0.0001
HLA sensitized pre-txp, n (%)	210 (24.9%)	178 (24.7%)	32 (26)%	0.735
Re-transplant, n (%)	70 (8.3%)	3 (8.8%)	7 (5.7%)	0.256
ABO, n (%)				
A	333 (39.5%)	279 (38.7%)	54 (43.9%)	0.28
B	108 (12.8%)	96 (13.3%)	12 (9.8%)	0.273
O	363 (43.1%)	313 (43.5%)	50 (40.7%)	0.559
AB	39 (4.6%)	32 (4.4%)	7 (5.7%)	0.543
Donor				
Donor age (year), Median (IQR)	39.5 (28.1-50.4)	40.2 (28.5-51.1)	35.8 (25.4-46.6)	0.0062
Deceased donor, n (%)	605 (71.8%)	527 (73.2%)	78 (63.4%)	0.026
Living donor, related, n (%)	111 (13.2%)	96 (13.3%)	15 (12.2%)	0.73
Living donor, unrelated, n (%)	127 (15.1%)	97 (13.5%)	30 (24.4%)	0.002
Post-Transplant				
Follow-up time (year), Median (IQR)	3.19 (1.83-5.74)	2.86 (1.69-4.85)	6.42 (3.89-8.37)	< 0.0001
dnDSA detection (year), Median (IQR)	N/A	N/A	2.15 (1.23-3.47)	
dnDSA Locus, n (%)				
Class I only	N/A	N/A	11 (8.9%)	
Class II only	N/A	N/A	82 (66.7%)	
Class I+II	N/A	N/A	30 (24.4%)	
A	N/A	N/A	30 (24.4%)	
B	N/A	N/A	19 (15.4%)	
C	N/A	N/A	15 (12.2%)	
DR	N/A	N/A	35 (28.5%)	
DQ	N/A	N/A	103 (83.7%)	

Of the 123 dnDSA+ patients, 11 (8.9%) developed dnDSA only to HLA Class I antigens, 82 (66.7%) to Class II only, and 30 recipients (24.4%) have both Class I and Class II dnDSAs. Expectedly, DQB1 is the most prevalent dnDSA locus, with 103 patients positive for DQ-dnDSAs (83.7% of dnDSA+ group). The median follow-up time of the study was 3.19 years, with significantly longer follow-up time in dnDSA+ patients (6.42 years) than dnDSA- subjects (2.86 years). The median onset of dnDSA detection was 2.15 years post-transplant.

### 3.2 Validation of high resolution typing imputation

As shown in **Supplementary Figure 1**, imputation of over 85% of HLA-A, C, and DQB1 alleles resulted in accurate high resolution types. There was a 79% agreement between imputed and actual HLA-B alleles, whereas the imputation of DRB1 alleles resulted in the lowest accuracy percentage (66%). Nevertheless, the correlation between all of the HLA-A/B/C and DR/DQ molecular mismatch scores derived from the actual alleles and imputed high resolution typing are significantly strong (r_s_ > 0.9 and p <0.0001 for all of the scores), suggesting that the impact of high resolution typing imputation on molecular mismatch assessment is insignificant.

### 3.3 B cell and T cell molecular mismatches are elevated in dnDSA+ recipients

**Figure 2:**
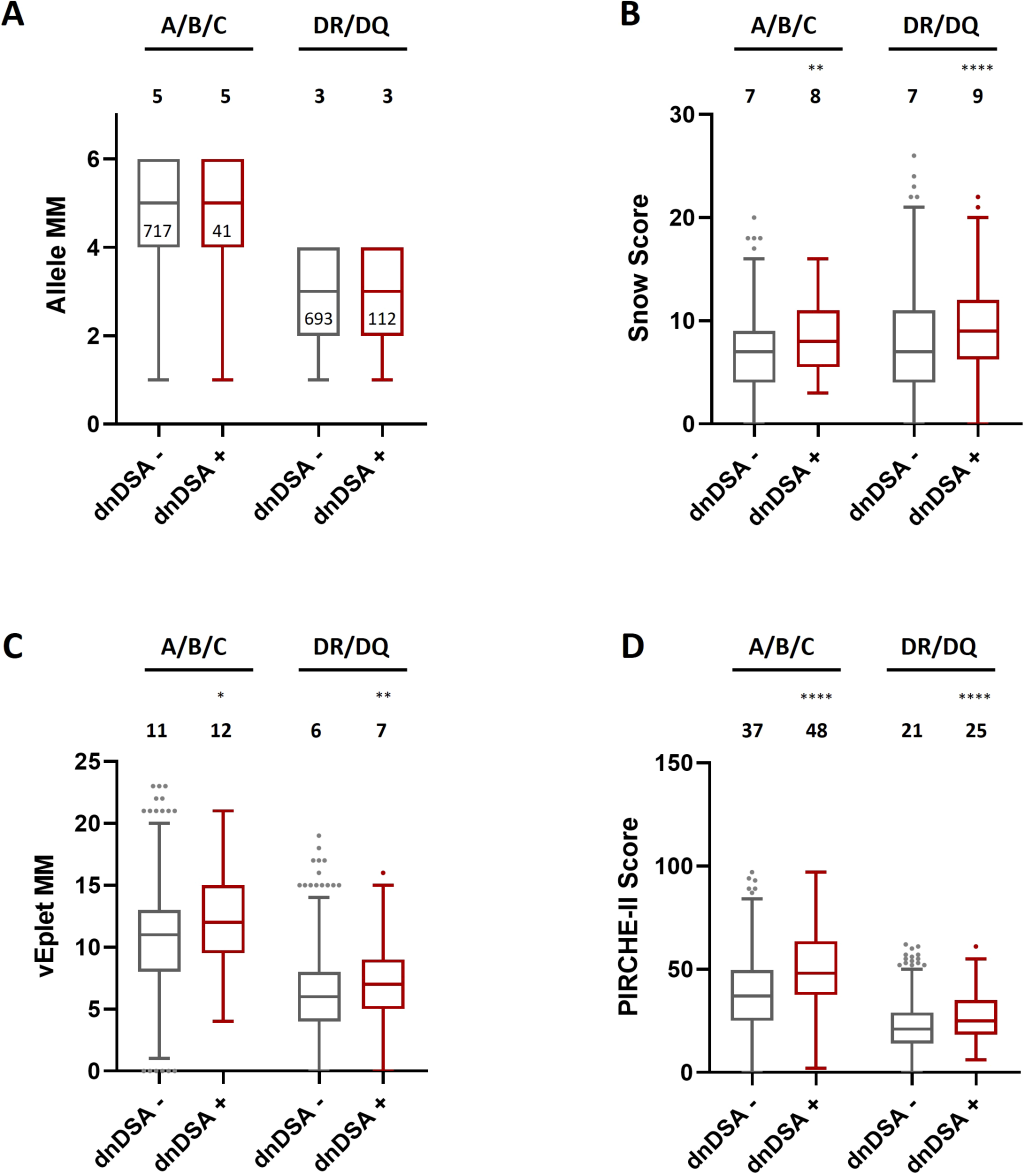
HLA Class I and combined DRB1/DQB1 allele mismatches **(A)**, Snow scores **(B)**, verified eplet mismatches **(C)**, and PIRCHE-II scores **(D)** in dnDSA- and dnDSA+ recipients after excluding the transplant pairs who are allele-matched at the respective loci. Box and Whisker plots represent the median (line in the middle of the box), 1^st^ to 3^rd^ quartile (box), and 1.5x interquartile range (whisker). Outliers are depicted as dots outside the whiskers. Median values are indicated above the plots. Numbers of patients in each group are shown within the boxes in plot **(A)**. *p < 0.05, **p < 0.01, ****p < 0.0001.

**Figure 3:**
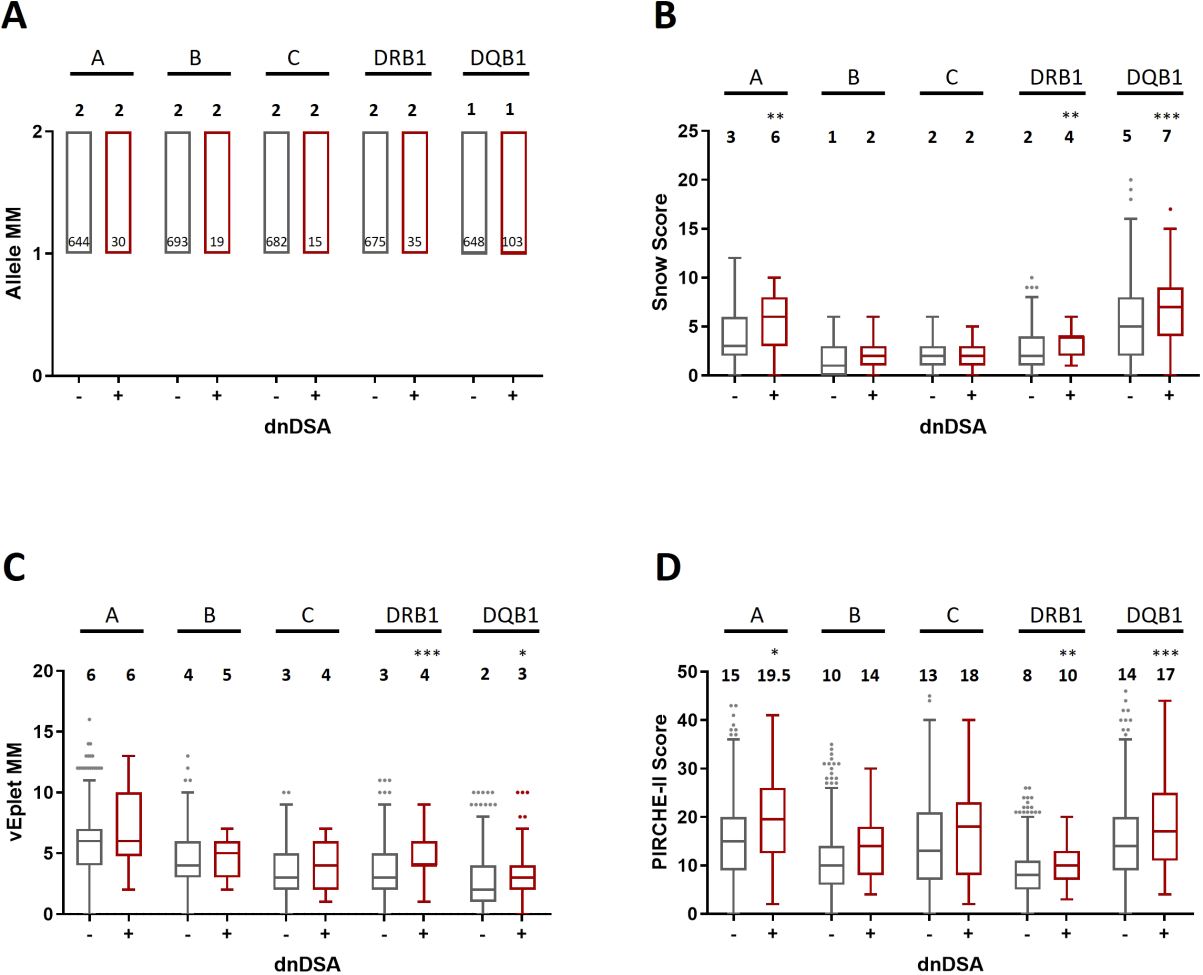
Allele mismatches **(A)**, Snow scores **(B)**, verified eplet mismatches **(C)**, and PIRCHE-II scores **(D)** for individual HLA-A, B, C, DRB1, and DQB1 loci in dnDSA- and dnDSA+ patients, excluding allele-matched pairs at the respective locus. Box and Whisker plots represent the median (line in the middle of the box), 1^st^ to 3^rd^ quartile (box), and 1.5x interquartile range (whisker). Outliers are depicted as dots outside the whiskers. Median values are indicated above the plots. Numbers of patients in each group are shown within the boxes in plot **(A)**. *p < 0.05, **p < 0.01, ***p < 0.001.

As shown in **Figure 2**; **Supplementary Table 1**, after excluding the transplant pairs who were allele-matched at HLA-A, B, and C loci, we found that although the median numbers of Class I allele mismatches between the dnDSA- (n= 717) and dnDSA+ (n= 41) groups are comparable (**Figure 2A**), the median sums of molecular mismatch scores for Class I loci, including Snow, verified eplet, and PIRCHE-II, are all higher in the Class I dnDSA+ recipients (**Figures 2B–D**). Similarly, in the recipients who have at least one allele mismatch at DRB1 or DQB1 locus (n= 805; dnDSA- n= 693, dnDSA+ n= 112), the medians of combined DRB1/DQB1 B cell and T cell epitope mismatches are also significantly elevated in dnDSA+ individuals, while the medians of allele mismatch numbers are equivalent between the groups. Furthermore, when evaluating individual HLA loci, the molecular mismatch scores at DRB1 and DQB1 are remarkably increased in dnDSA+ recipients (**Figure 3** and **Supplementary Table 2**).

**Figure 4:**
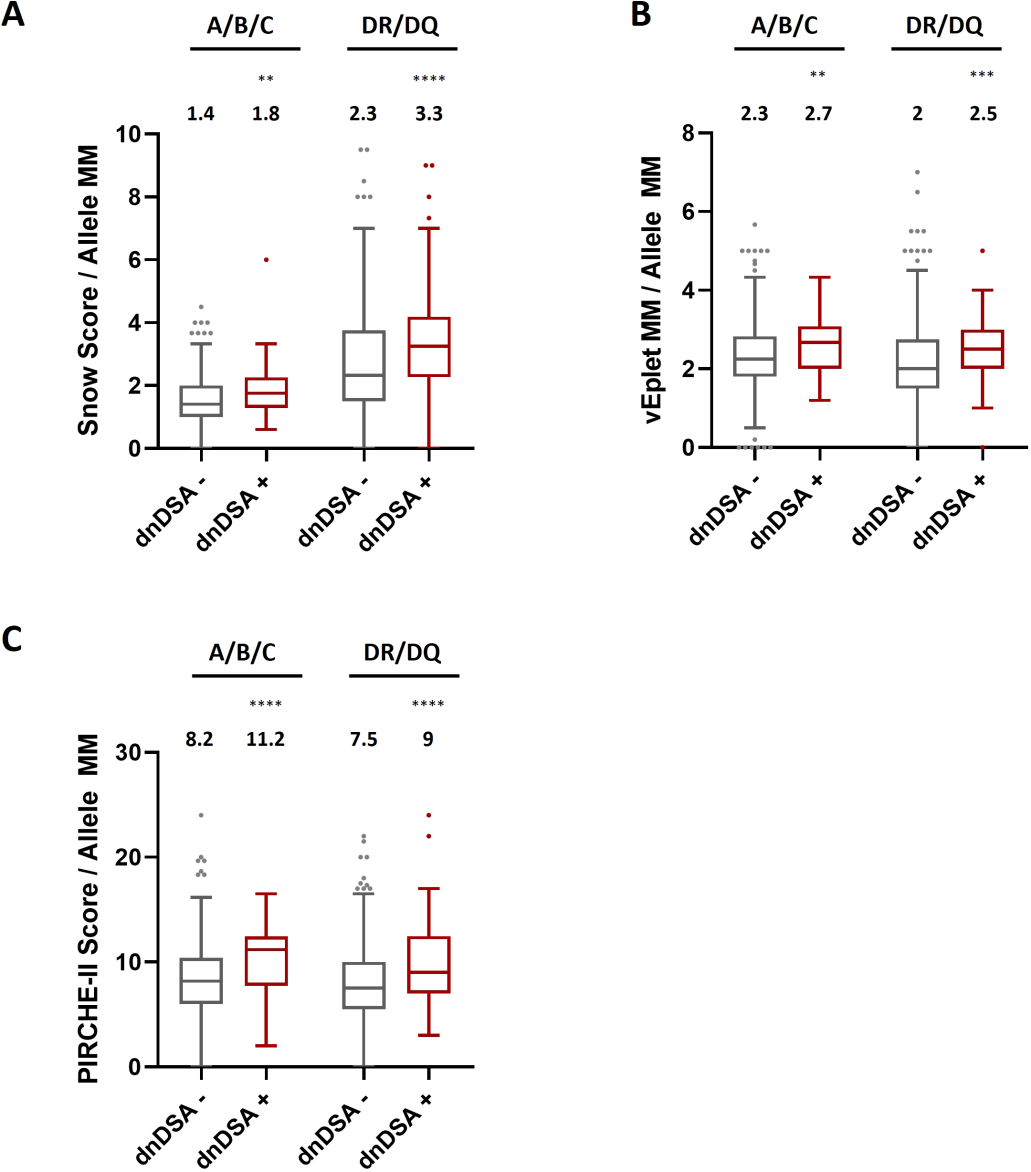
HLA Class I and combined DRB1/DQB1 Snow scores **(A)**, verified eplet mismatches **(B)**, and PIRCHE-II scores **(C)** in dnDSA- and dnDSA+ recipients after normalization by the numbers of allele mismatches at the respective loci for individual study subjects. Box and Whisker plots represent the median (line in the middle of the box), 1^st^ to 3^rd^ quartile (box), and 1.5x interquartile range (whisker). Outliers are depicted as dots outside the whiskers. Median values are indicated above the plots. **p < 0.01, ***p < 0.001, ****p < 0.0001.

Next, to further ensure that the dissimilarities of epitope mismatches between dnDSA+ and dnDSA- recipients were not a result of differential allele mismatch numbers in individual patients, we divided the molecular mismatch scores by the numbers of allele mismatches at the respective loci of analysis for each study subject. Our data showed that after normalization by the allele mismatch numbers, the median molecular mismatch scores remained remarkably higher in the dnDSA+ group for both Class I and DRB1/DQB1 loci (**Figure 4** and **Supplementary Table 3**). These results confirmed that the increased molecular mismatches in dnDSA+ patients were independent of allele mismatches. Similarly, molecular mismatch scores are also significantly elevated in dnDSA+ patients after normalization by the numbers of antigen mismatches at the respective loci, with the exception of A/B/C vEplet MMs which is increased but not reaching statistical significance (p= 0.051) (**Supplementary Figure 2**, **Supplementary Table 4**).

### 3.4 Correlation between molecular mismatch scores

In concordance with previous publication (9), in this study cohort we observed strong correlation between the two B cell epitope analysis algorithms, Snow and verified eplet mismatches (Class I: Spearman’s rank correlation coefficient r_s_ = 0.754, p < 0.0001; DRB1+DQB1: r_s_ = 0.807, p < 0.0001), as well as moderate correlation between B cell and T cell epitope mismatches for Class I (Snow vs. PIRCHE-II r_s_ = 0.598, p < 0.0001; vEplet vs. PIRCHE-II r_s_ = 0.681, p < 0.0001) and DRB1+DQB1 loci (Snow vs. PIRCHE-II r_s_ = 0.689, p < 0.0001; vEplet vs. PIRCHE-II r_s_ = 0.692, p < 0.0001) (**Table 2**).

**Table 2:** Correlation between Snow, verified eplet, and PIRCHE-II scores.

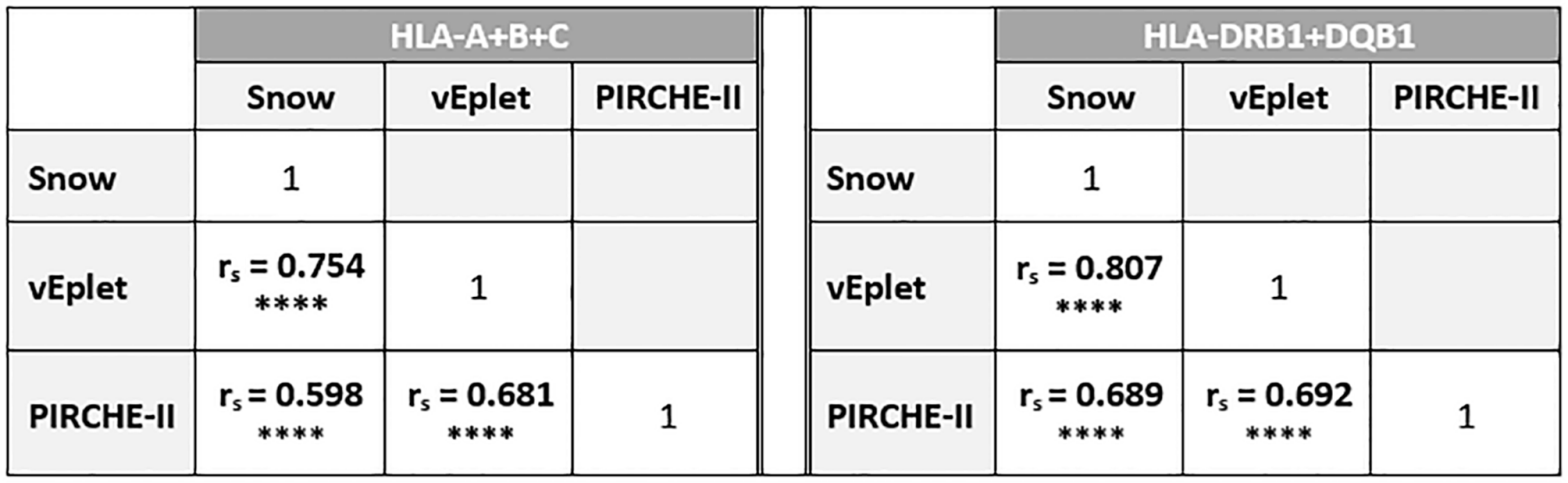

r_s_ = Spearman’s rank correlation coefficient. **** p<0.0001.

### 3.5 Association of elevated molecular mismatches with dnDSA development

**Figure 5:**
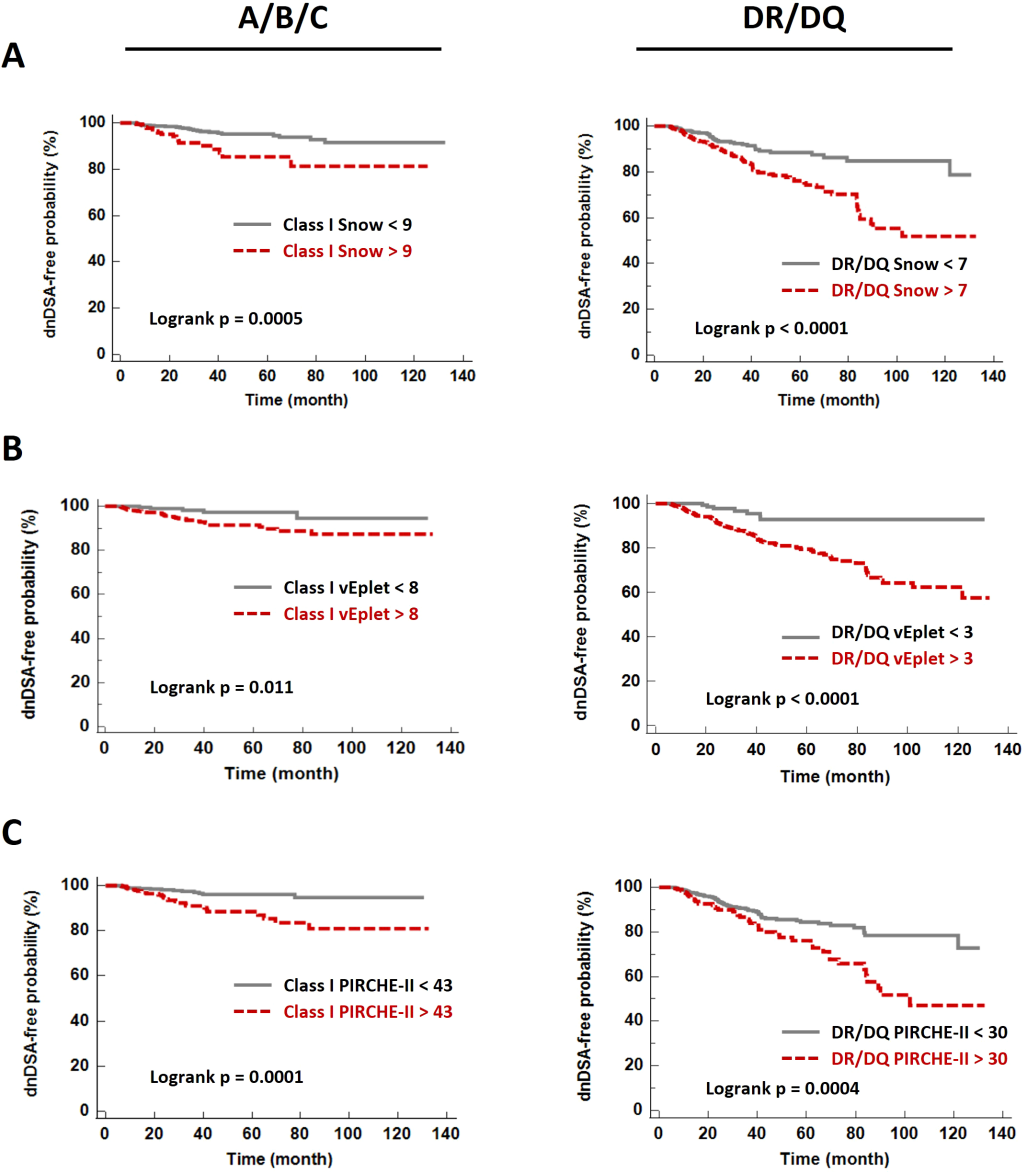
*De novo* DSA-free probabilities in patients with Snow **(A)**, verified eplet mismatch **(B)**, and PIRCHE-II **(C)** scores lower (grey solid lines) and greater (red dashed lines) than the ROC-defined thresholds.

ROC (Receiver Operating Characteristic) analyses identified that Class I Snow score > 9, verified eplet mismatch > 8, and PIRCHE-II score > 43 were associated with HLA-A/B/C dnDSA formation, while DRB1+DQB1 Snow > 7, verified eplet mismatch > 3, and PIRCHE-II score > 30 correlated with the development of DRB1 and/or DQB1 dnDSA (**Table 3**, **Supplementary Figure 3**). Accordingly, molecular mismatch scores greater than the ROC-defined thresholds were associated with significantly lower dnDSA-free probabilities in Kaplan-Meier analysis (**Figure 5**).

**Table 3:** Thresholds of Snow, vEplet, and PIRCHE-II scores identified by ROC curve analysis to be associated with Class I and DRB1/DQB1 dnDSA formation.

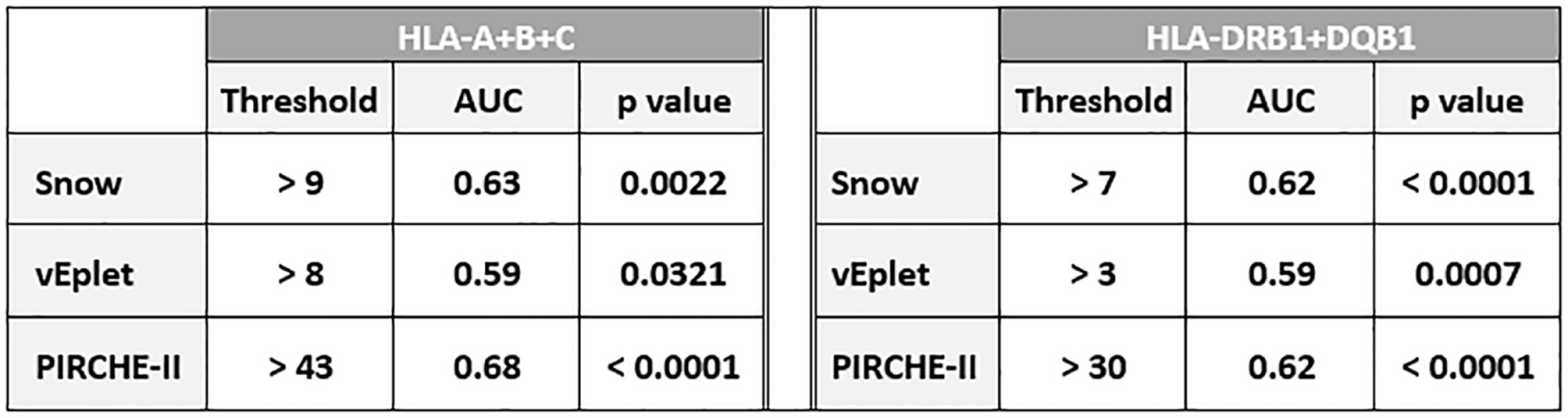

AUC, Area Under the Curve.

### 3.6 Synergistic effect of B cell and T cell epitope mismatches on the occurrence of dnDSA

**Figure 6:**
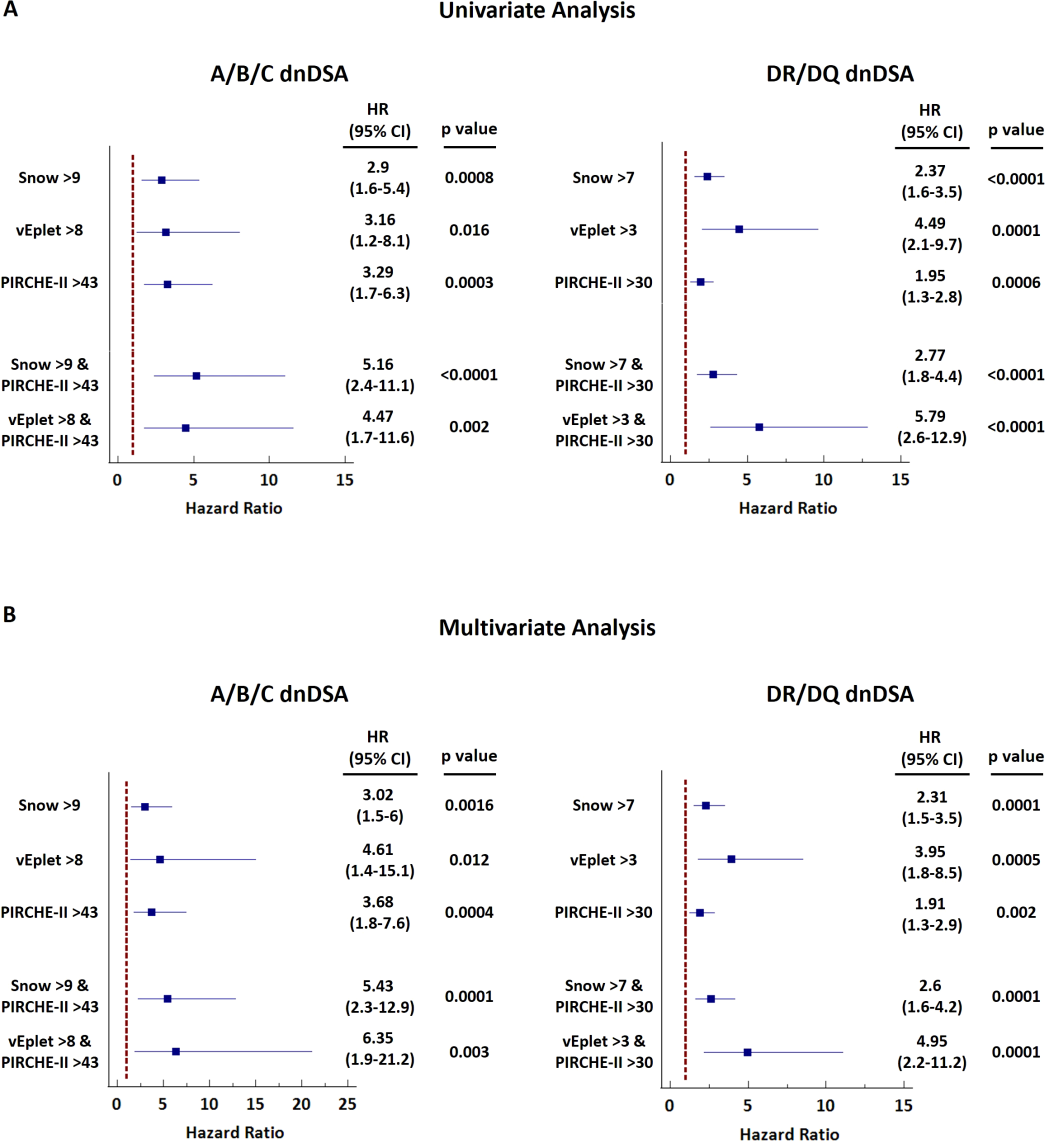
Forest plots of univariate **(A)** and multivariate **(B)** Cox regression analyses for dnDSA occurrence with single or combined B cell and T cell molecular mismatch scores. Multivariate model was adjusted for recipient age at transplant, pre-transplant CPRA (%), gender, pediatric patient, repeat transplantation, donor age, and donor type.

Upon categorizing the recipients into three subgroups based on B cell and T cell epitope mismatch scores – both scores below the ROC-defined cutoffs, one of the scores greater than the cutoffs, and both scores greater than the cutoffs, we found that individual, as well as combined B cell and T cell molecular mismatch scores greater than the ROC-defined cutoffs are significantly associated with increased dnDSA hazard ratios (HR) by univariate Cox regression analysis (**Figure 6A**, **Supplementary Table 5**), and multivariate Cox model adjusted for recipient age at transplant, pre-transplant CPRA (%), gender, pediatric patient, repeat transplantation, donor age, and donor type (**Figure 6B**, **Supplementary Table 5**). Of note, HRs of both Snow and PIRCHE-II scores greater than cutoffs (A+B+C Univariate: 5.16, Multivariate: 5.43; DR+DQ Univariate: 2.77, Multivariate: 2.6) are higher than the HRs of individual Snow > cutoff (A+B+C Univariate: 2.9, Multivariate: 3.02; DR+DQ Univariate: 2.37, Multivariate: 2.31) and PIRCHE-II > cutoff (A+B+C Univariate: 3.29, Multivariate: 3.68; DR+DQ Univariate: 1.95, Multivariate: 1.91). Similar trend is also observed with vEplet and PIRCHE-II – HRs of both scores greater than cutoffs (A+B+C Univariate: 4.47, Multivariate: 6.35; DR+DQ Univariate: 5.79, Multivariate: 4.95) are higher than vEplet > cutoff (A+B+C Univariate: 3.16, Multivariate: 4.61; DR+DQ Univariate: 4.49, Multivariate: 3.95) and PIRCHE-II > cutoff.

**Figure 7:**
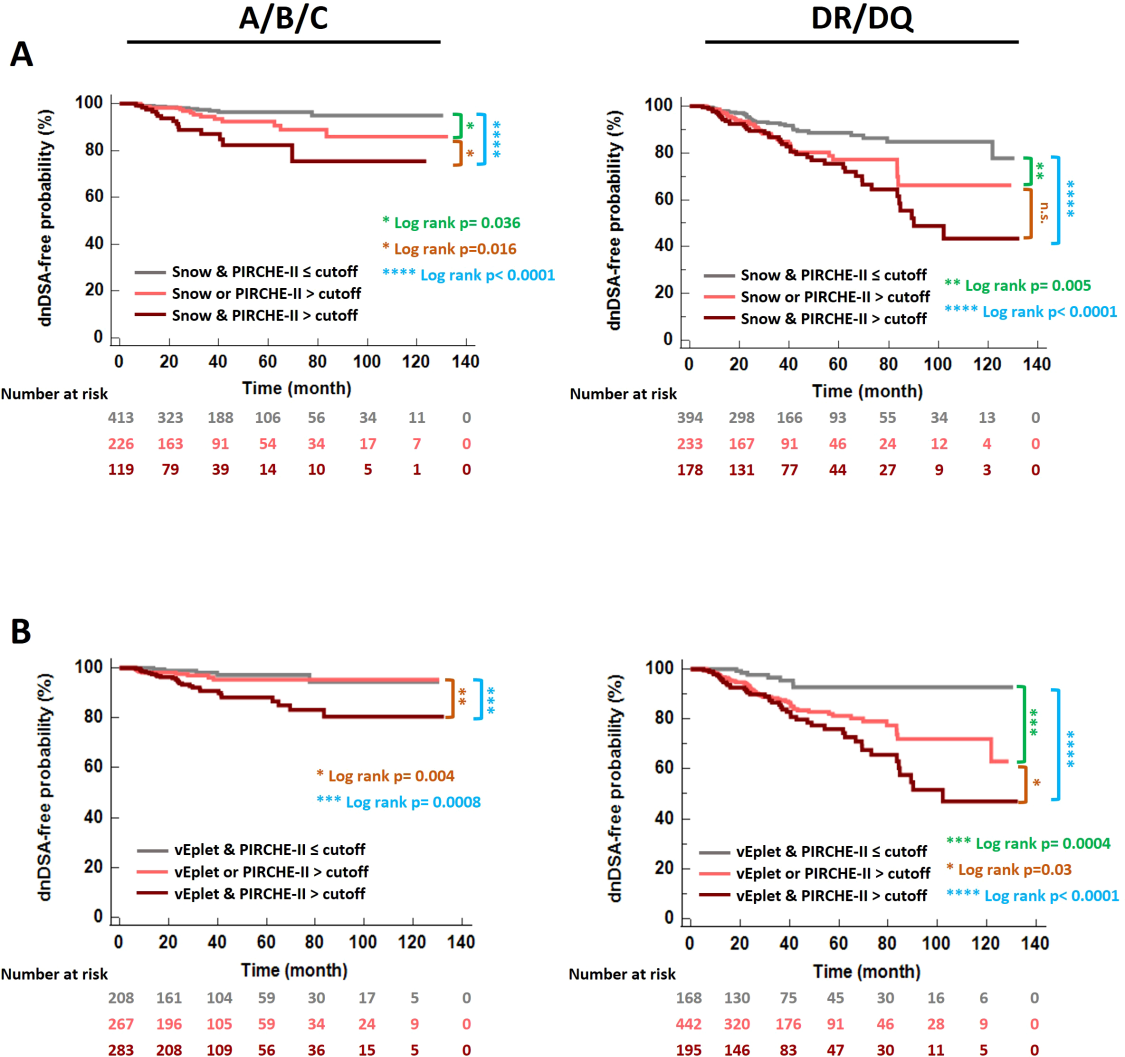
*De novo* DSA-free probabilities in patients categorized according to B cell and T cell epitope mismatch loads – both scores below the ROC-defined cutoffs (grey), one of the scores greater than the cutoff (light pink), and both scores greater than the cutoffs (dark pink). **(A)** Snow and PIRCHE-II scores, **(B)** verified eplet and PIRCHE-II scores. Log rank p values were indicated with asterisks in different colors: Green- Both scores ≤ cutoffs vs. one of the scores > cutoff. Blue- Both scores ≤ cutoffs vs. both scores > cutoffs. Orange- One of the scores > cutoff vs. both scores > cutoffs.

Correspondingly, Kaplan-Meier analysis revealed that the subgroup of patients who scored high by both B cell and T cell epitope mismatch algorithms (dark pink lines in **Figure 7**) demonstrated the lowest dnDSA-free probabilities, compared to recipients with one (light pink lines in **Figure 7**) or both scores below the cutoffs (grey lines in **Figure 7**). These results demonstrated a potentially more refined stratification for the risk of dnDSA development utilizing a combination of B cell and T cell molecular mismatch assessment.

### 3.7 Validation of dnDSA risk stratification using defined molecular mismatch thresholds

To validate the association of dnDSA formation and elevated molecular mismatch scores, we evaluated HLA-A/B/C and DR/DQ Snow, vEplet, and PIRCHE-II scores of a separate cohort of 544 kidney transplant patients and found that the dnDSA+ group has significantly higher percentages of patients with molecular mismatch scores greater than the defined cutoffs, compared to the patients without dnDSA (**Supplementary Figure 4A**). Furthermore, the significant decrease of dnDSA-free probability in the patients with both B cell and T cell epitope mismatches greater than the thresholds is also confirmed in the validation cohort (**Supplementary Figure 4B**).

## 4 Discussion

In this retrospective study, we evaluated the correlations between HLA epitope mismatches assessed by three algorithms - Snow, HLAMatchmaker, and an updated version of PIRCHE-II, with *de novo* DSA development in a cohort of 843 kidney transplant recipients. Our results demonstrated that B cell and T cell epitope mismatches are remarkably increased in dnDSA+ recipients, and importantly, elevated numbers of epitope mismatches are significantly predictive of dnDSA occurrence in kidney transplantation.

To our knowledge, this report is the first to evaluate the utility of the newly developed Snow algorithm for the prediction of dnDSA using local kidney transplant data. Snow takes both the solvent-accessible surface area (Snowflake (7)), and amino acid protrusion (Snowball (8)) of individual HLA alleles into consideration and provides a refined measure to identify potential antibody epitopes. In agreement with previous publication (9), we observed strong correlation between Snow scores and verified eplet mismatches in our patient cohort (**Table 2**).

One of the strengths of our study lies in the painstaking approach to data analysis. We utilized three HLA molecular mismatch assessment algorithms and compared B cell and T cell epitope mismatches at individual HLA-A, B, C, DRB1, DQB1 loci, or combined Class I and DRB1/DQB1 loci after excluding any transplant pairs who are allele-matched at the locus/loci of analysis to avoid misrepresentation of the molecular mismatch scores. With this refined analysis scheme, we observed strong correlations of individual and combined DRB1/DQB1 epitope mismatch loads with the occurrence of DRB1 and/or DQB1 dnDSA (**Figures 2** and **3**, **Supplementary Tables 1** and **Supplementary Table 2**), which is consistent with the findings demonstrated by several other groups (4, 13, 17–19). On the other hand, although the lack of clear correlation between Class I epitope mismatches and the risks for inferior kidney transplant outcomes has been presented in a number of studies (16, 18, 20, 21), our results showed that the combined HLA-A/B/C Snow, vEplet, and PIRCHE-II scores are all distinctly greater in the Class I dnDSA+ patients, compared to the dnDSA- group (**Figure 2**, **Supplementary Table 1**). Furthermore, our study assessed epitope mismatches after adjusting for allele mismatch numbers in each patient. This analysis approach avoids inaccurate interpretation of the molecular mismatch scores when there are differential numbers of allele mismatches among the study subjects. Our data demonstrated that Snow, vEplet, and PIRCHE-II scores per mismatched allele are all significantly elevated in the dnDSA+ group (**Figure 4**, **Supplementary Table 3**), verifying that B cell and T cell epitope mismatches are indeed strong contributors to the development of Class I and DRB1/DQB1 *de novo* DSAs independent of allele mismatch numbers.

During a typical immune response to alloantigens in transplantation, donor-specific naïve B cells are activated upon the recognition of alloantigens via their surface B cell receptor (BCR). Also through BCR, B cells endocytose donor antigens, degrade them in the lysosomes, and present donor-derived peptides in the form of MHC class II-peptide complexes on their surfaces. The primed B cells then migrate to the border of the T cell zone in the secondary lymphoid organs where they interact with CD4^+^ helper T cells that carry cognate T cell receptor (TCR) specific for the presented donor-derived peptides. This interaction leads to the activation of the helper T cells, which in turn deliver stimulation signals through CD40/CD40L binding and the release of cytokines that trigger the expansion and differentiation of B cells into antibody-secreting plasma cells. As the establishment of humoral alloimmune responses requires the interaction between B and T cells, it is imperative to take both B cell and T cell epitopes into account when evaluating the level of compatibility between transplant donors and recipients. Supporting this notion, our results distinctly showed that the subgroup of patients who score high by both T cell (PIRCHE-II) and B cell (Snow or vEplet) molecular mismatch algorithms exhibited the lowest Class I and DRB1/DQB1 dnDSA-free probabilities, compared to patients with only one of the mismatch scores above the ROC-defined threshold (**Figure 7**). Furthermore, univariate and multivariate Cox regression also showed an increase of hazard ratios with the combined scores. Although verifying the improvement of dnDSA prediction with the combined B cell and T cell epitope mismatch scores in a larger cohort is warranted, our data highlight the potential of a more refined analysis approach utilizing a combination of B cell and T cell molecular mismatch assessment in donor selection and recipient immunological risk stratification, and calls for the development of computer algorithms that incorporate both T and B cell epitopes in the evaluation of recipient-donor histocompatibility.

Our study has several limitations. First, the study cohort is comprised of kidney recipients from four transplant centers, including one pediatric and three adult programs. As each center has specific protocols for immunosuppression regimens and patient management, the heterogeneity of the study subjects restricts the potential to further examine the immunogenicity of individual epitopes in this cohort. This limitation can be mitigated by using the “2MM1DSA” model conceptualized by Tambur et al. (22), where the immunogenicity of epitopes is evaluated in a patient-specific setting so that other factors that may influence immune responses in the study individuals can be controlled. Secondly, the calculation of epitope mismatches was based on high resolution typing imputed from the antigen level. The challenges of accurate HLA typing imputation, especially for non-Caucasian individuals, have been discussed in several reports (23, 24). We acknowledge that in addition to the intrinsic limitation of high resolution typing imputation, the lack of accurate ethnicity information for some of the study subjects may have further restricted our ability to infer their HLA genotype without error (25). Compared to the single imputation method employed in this study, a multiple imputation algorithm that calculates a “summed” epitope mismatch scores using weighted genotype combinations based on haplotype frequencies may improve the accuracy of epitope mismatch prediction (26, 27). Although we and others (28–31) have shown that there is reasonable concordance between molecular mismatch numbers calculated from imputed and high resolution typing (**Supplementary Figure 1**), we recognize the criticality of correct HLA allele information for the application of epitope matching assessment in the clinical settings. With the advent of novel NGS platforms, such as Nanopore technology, rapid high resolution typing of deceased donors will soon be achievable (32–34). Another limitation of this study is the lack of data on the clinical outcomes, such as ABMR, graft survival, and patient survival. It is well recognized that the presence of dnDSA is a strong risk factor for ABMR and inferior graft survival (35–37), nonetheless, the association of molecular mismatch loads with clinical outcomes in this study cohort remains to be identified. Moreover, while a comprehensive transplant compatibility evaluation requires patient and donor’s allele-level typing at all of the HLA loci, the calculation of T cell and B cell epitope mismatches in this study were only based on HLA-A, B, C, DRB1, and DQB1 loci due to insufficient information available on DRB345, DQA1, and DPA1/DPB1 typing. Along the same line, the T cell epitope mismatches presented by the PIRCHE-II scores in our study only represent the numbers of donor-derived peptides presented by recipient’s DRB1 antigens. Even though it is generally recognized that DQ and DP molecules have lower surface expression, further analyses that include peptides presented by recipient DQ and DP proteins, as well as incorporate alleles from all HLA loci, are required to verify the observations in this study. Lastly, evaluation of combined Class I and DR+DQ molecular mismatch scores does not allow for risk assessment for dnDSA at individual HLA locus, which may have differential clinical significance depending on their expression levels.

In conclusion, in the current study we utilized three HLA molecular mismatch assessment algorithms and evaluated the correlation of B cell and T cell epitope mismatch loads with *de novo* DSA development in a large kidney transplant cohort. We demonstrated a significant association of Snow, verified eplet, and PIRCHE-II scores with dnDSA at HLA Class I and DRB1/DQB1 loci, independently and in synergy. Our data highlight the value of applying HLA epitope mismatch evaluation in living donor selection and immunological risk stratification to improve transplant outcomes.

## Data Availability

The raw data supporting the conclusions of this article will be made available by the authors, without undue reservation.
