# Supplementary material for: De Novo donor-specific anti-HLA antibody risk stratification in kidney transplantation using a combination of B cell and T cell molecular mismatch assessment
Source: Front Immunol. 2025 Feb 25;16:1508796. doi: 10.3389/fimmu.2025.1508796 (PMC11893832; doi:10.3389/fimmu.2025.1508796)
Supplement: Supplementary file 1 [file Presentation1.pptx]

## Slide 1
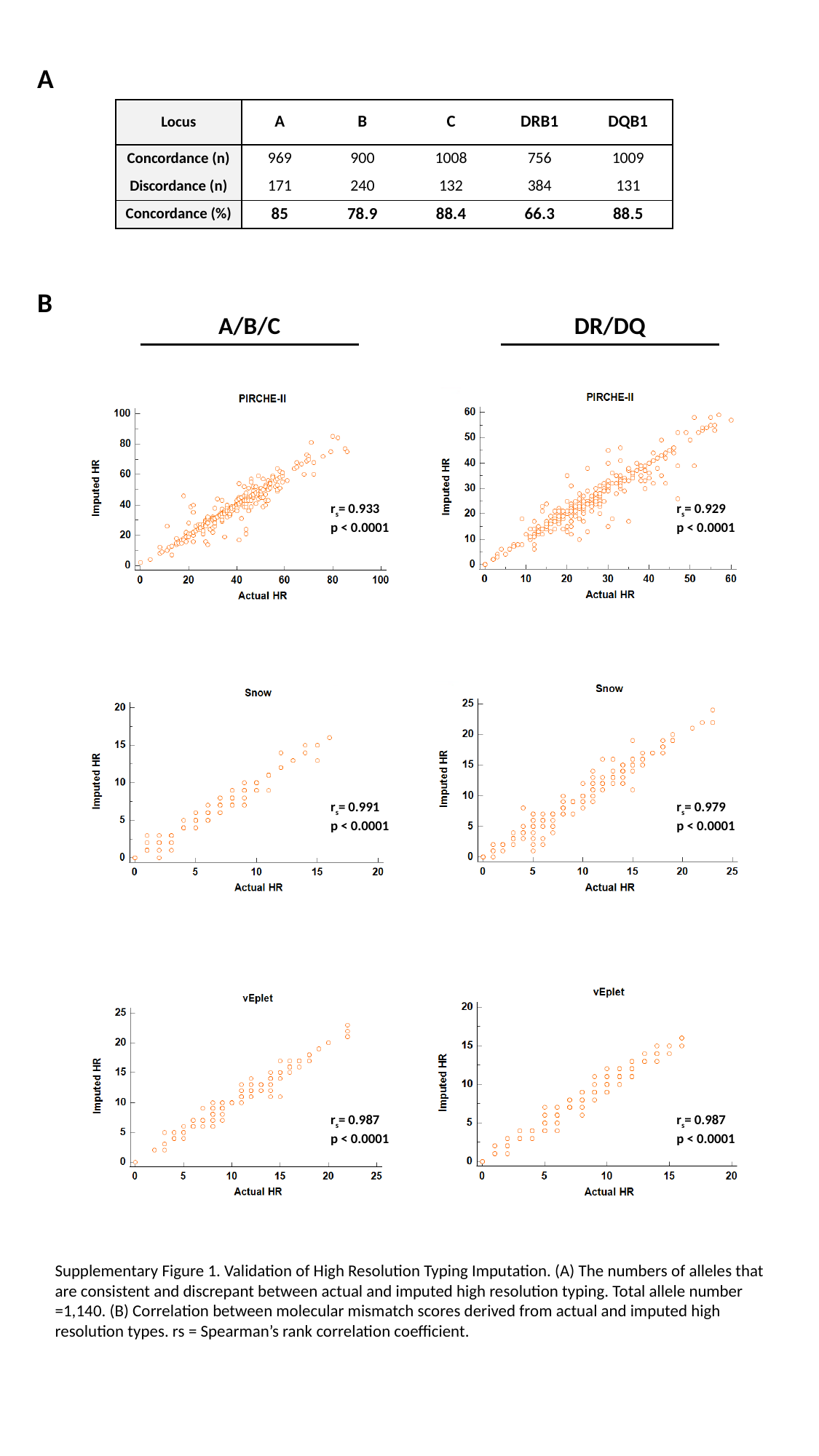

A
| Locus | A | B | C | DRB1 | DQB1 |
| --- | --- | --- | --- | --- | --- |
| Concordance (n) | 969 | 900 | 1008 | 756 | 1009 |
| Discordance (n) | 171 | 240 | 132 | 384 | 131 |
| Concordance (%) | 85 | 78.9 | 88.4 | 66.3 | 88.5 |
B
A/B/C
DR/DQ
rs= 0.933
p < 0.0001
rs= 0.929
p < 0.0001
rs= 0.991
p < 0.0001
rs= 0.979
p < 0.0001
rs= 0.987
p < 0.0001
rs= 0.987
p < 0.0001
Supplementary Figure 1. Validation of High Resolution Typing Imputation. (A) The numbers of alleles that are consistent and discrepant between actual and imputed high resolution typing. Total allele number =1,140. (B) Correlation between molecular mismatch scores derived from actual and imputed high resolution types. rs = Spearman’s rank correlation coefficient.

## Slide 2
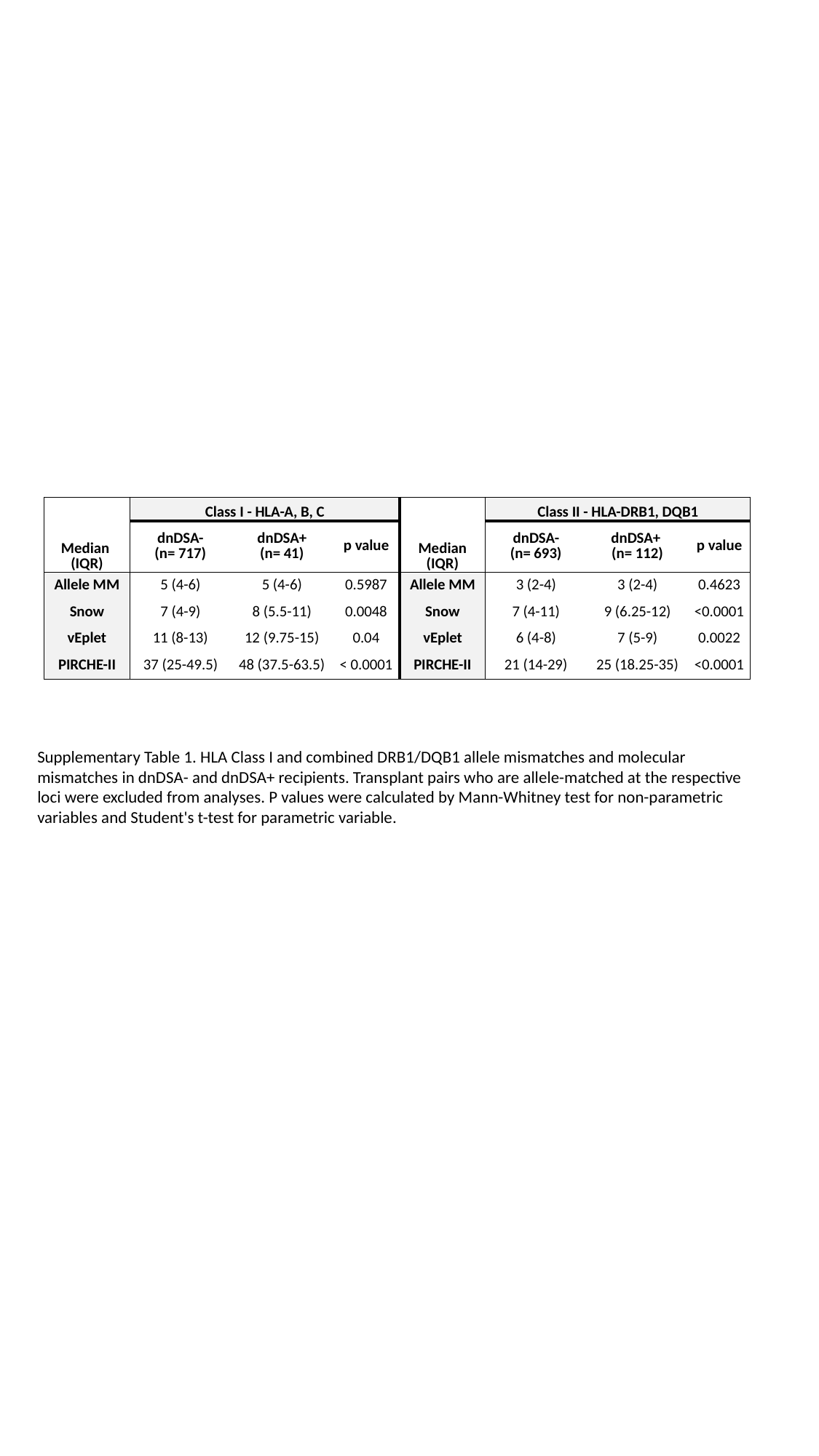

| | Class I - HLA-A, B, C | | | | Class II - HLA-DRB1, DQB1 | | |
| --- | --- | --- | --- | --- | --- | --- | --- |
| Median (IQR) | dnDSA- (n= 717) | dnDSA+ (n= 41) | p value | Median (IQR) | dnDSA- (n= 693) | dnDSA+ (n= 112) | p value |
| Allele MM | 5 (4-6) | 5 (4-6) | 0.5987 | Allele MM | 3 (2-4) | 3 (2-4) | 0.4623 |
| Snow | 7 (4-9) | 8 (5.5-11) | 0.0048 | Snow | 7 (4-11) | 9 (6.25-12) | <0.0001 |
| vEplet | 11 (8-13) | 12 (9.75-15) | 0.04 | vEplet | 6 (4-8) | 7 (5-9) | 0.0022 |
| PIRCHE-II | 37 (25-49.5) | 48 (37.5-63.5) | < 0.0001 | PIRCHE-II | 21 (14-29) | 25 (18.25-35) | <0.0001 |
Supplementary Table 1. HLA Class I and combined DRB1/DQB1 allele mismatches and molecular mismatches in dnDSA- and dnDSA+ recipients. Transplant pairs who are allele-matched at the respective loci were excluded from analyses. P values were calculated by Mann-Whitney test for non-parametric variables and Student's t-test for parametric variable.

## Slide 3
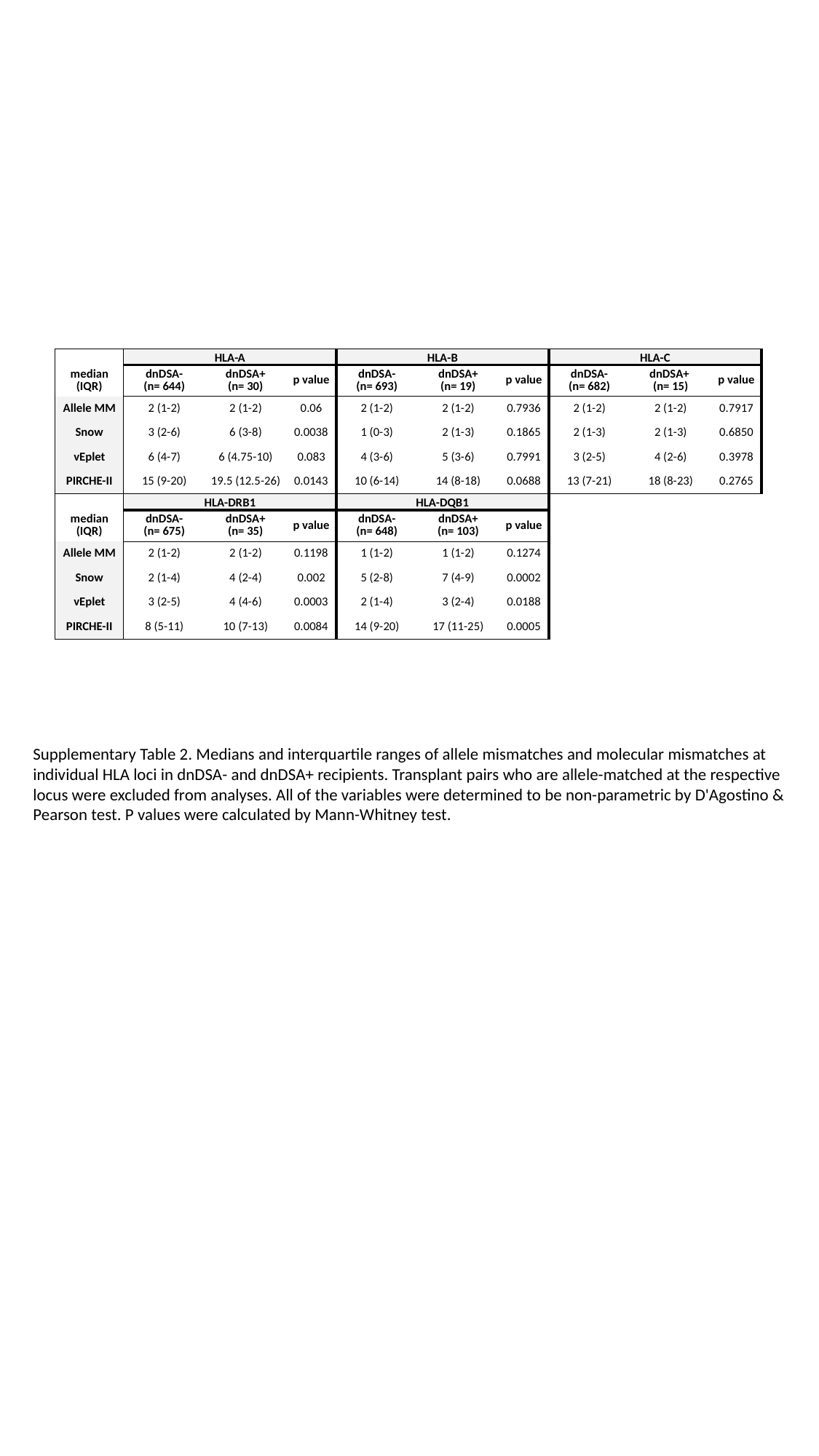

| | HLA-A | | | HLA-B | | | HLA-C | | |
| --- | --- | --- | --- | --- | --- | --- | --- | --- | --- |
| median (IQR) | dnDSA- (n= 644) | dnDSA+ (n= 30) | p value | dnDSA- (n= 693) | dnDSA+ (n= 19) | p value | dnDSA- (n= 682) | dnDSA+ (n= 15) | p value |
| Allele MM | 2 (1-2) | 2 (1-2) | 0.06 | 2 (1-2) | 2 (1-2) | 0.7936 | 2 (1-2) | 2 (1-2) | 0.7917 |
| Snow | 3 (2-6) | 6 (3-8) | 0.0038 | 1 (0-3) | 2 (1-3) | 0.1865 | 2 (1-3) | 2 (1-3) | 0.6850 |
| vEplet | 6 (4-7) | 6 (4.75-10) | 0.083 | 4 (3-6) | 5 (3-6) | 0.7991 | 3 (2-5) | 4 (2-6) | 0.3978 |
| PIRCHE-II | 15 (9-20) | 19.5 (12.5-26) | 0.0143 | 10 (6-14) | 14 (8-18) | 0.0688 | 13 (7-21) | 18 (8-23) | 0.2765 |
| | HLA-DRB1 | | | HLA-DQB1 | | | | | |
| median (IQR) | dnDSA- (n= 675) | dnDSA+ (n= 35) | p value | dnDSA- (n= 648) | dnDSA+ (n= 103) | p value | | | |
| Allele MM | 2 (1-2) | 2 (1-2) | 0.1198 | 1 (1-2) | 1 (1-2) | 0.1274 | | | |
| Snow | 2 (1-4) | 4 (2-4) | 0.002 | 5 (2-8) | 7 (4-9) | 0.0002 | | | |
| vEplet | 3 (2-5) | 4 (4-6) | 0.0003 | 2 (1-4) | 3 (2-4) | 0.0188 | | | |
| PIRCHE-II | 8 (5-11) | 10 (7-13) | 0.0084 | 14 (9-20) | 17 (11-25) | 0.0005 | | | |
Supplementary Table 2. Medians and interquartile ranges of allele mismatches and molecular mismatches at individual HLA loci in dnDSA- and dnDSA+ recipients. Transplant pairs who are allele-matched at the respective locus were excluded from analyses. All of the variables were determined to be non-parametric by D'Agostino & Pearson test. P values were calculated by Mann-Whitney test.

## Slide 4
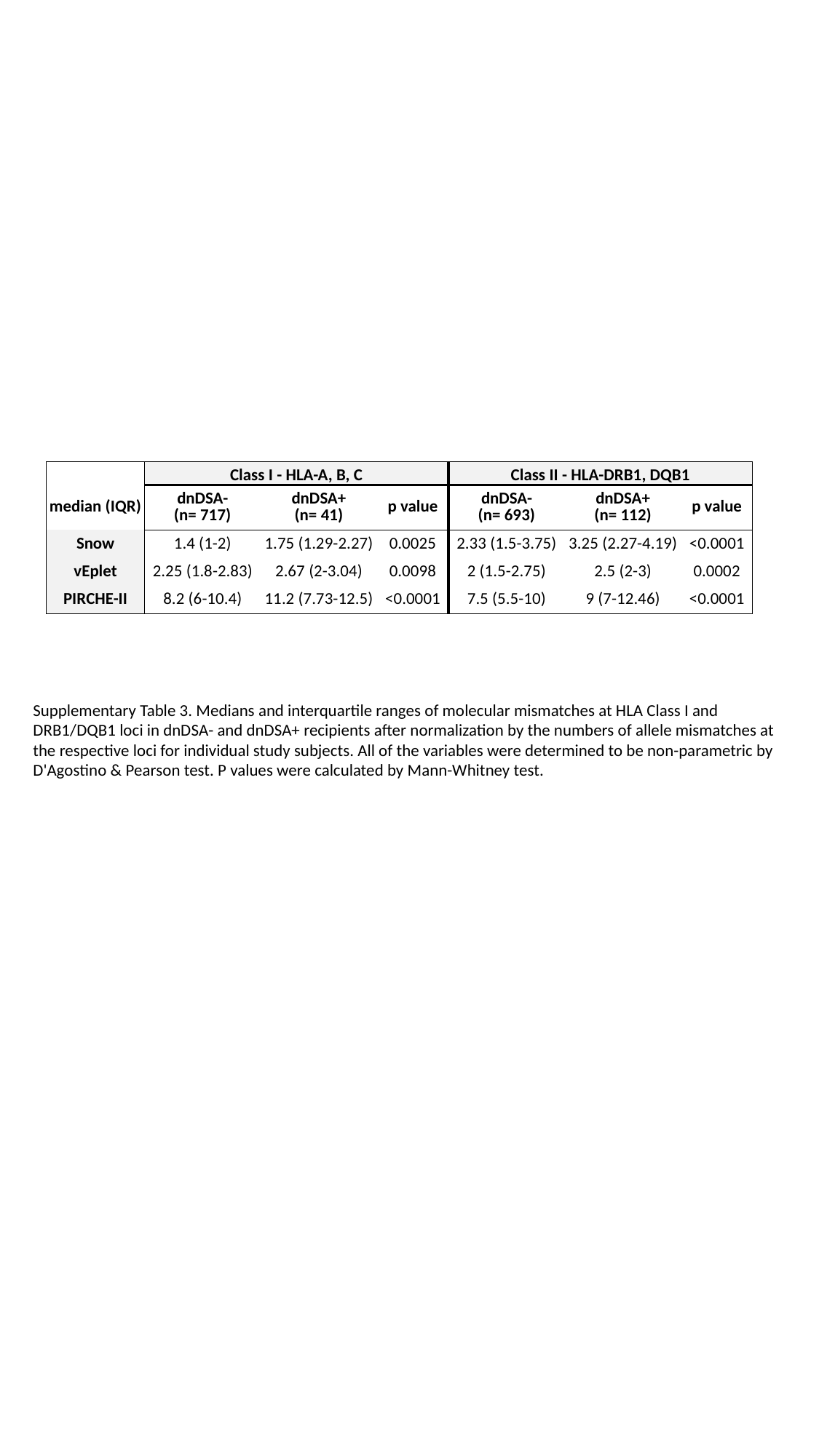

| | Class I - HLA-A, B, C | | | Class II - HLA-DRB1, DQB1 | | |
| --- | --- | --- | --- | --- | --- | --- |
| median (IQR) | dnDSA- (n= 717) | dnDSA+ (n= 41) | p value | dnDSA- (n= 693) | dnDSA+ (n= 112) | p value |
| Snow | 1.4 (1-2) | 1.75 (1.29-2.27) | 0.0025 | 2.33 (1.5-3.75) | 3.25 (2.27-4.19) | <0.0001 |
| vEplet | 2.25 (1.8-2.83) | 2.67 (2-3.04) | 0.0098 | 2 (1.5-2.75) | 2.5 (2-3) | 0.0002 |
| PIRCHE-II | 8.2 (6-10.4) | 11.2 (7.73-12.5) | <0.0001 | 7.5 (5.5-10) | 9 (7-12.46) | <0.0001 |
Supplementary Table 3. Medians and interquartile ranges of molecular mismatches at HLA Class I and DRB1/DQB1 loci in dnDSA- and dnDSA+ recipients after normalization by the numbers of allele mismatches at the respective loci for individual study subjects. All of the variables were determined to be non-parametric by D'Agostino & Pearson test. P values were calculated by Mann-Whitney test.

## Slide 5
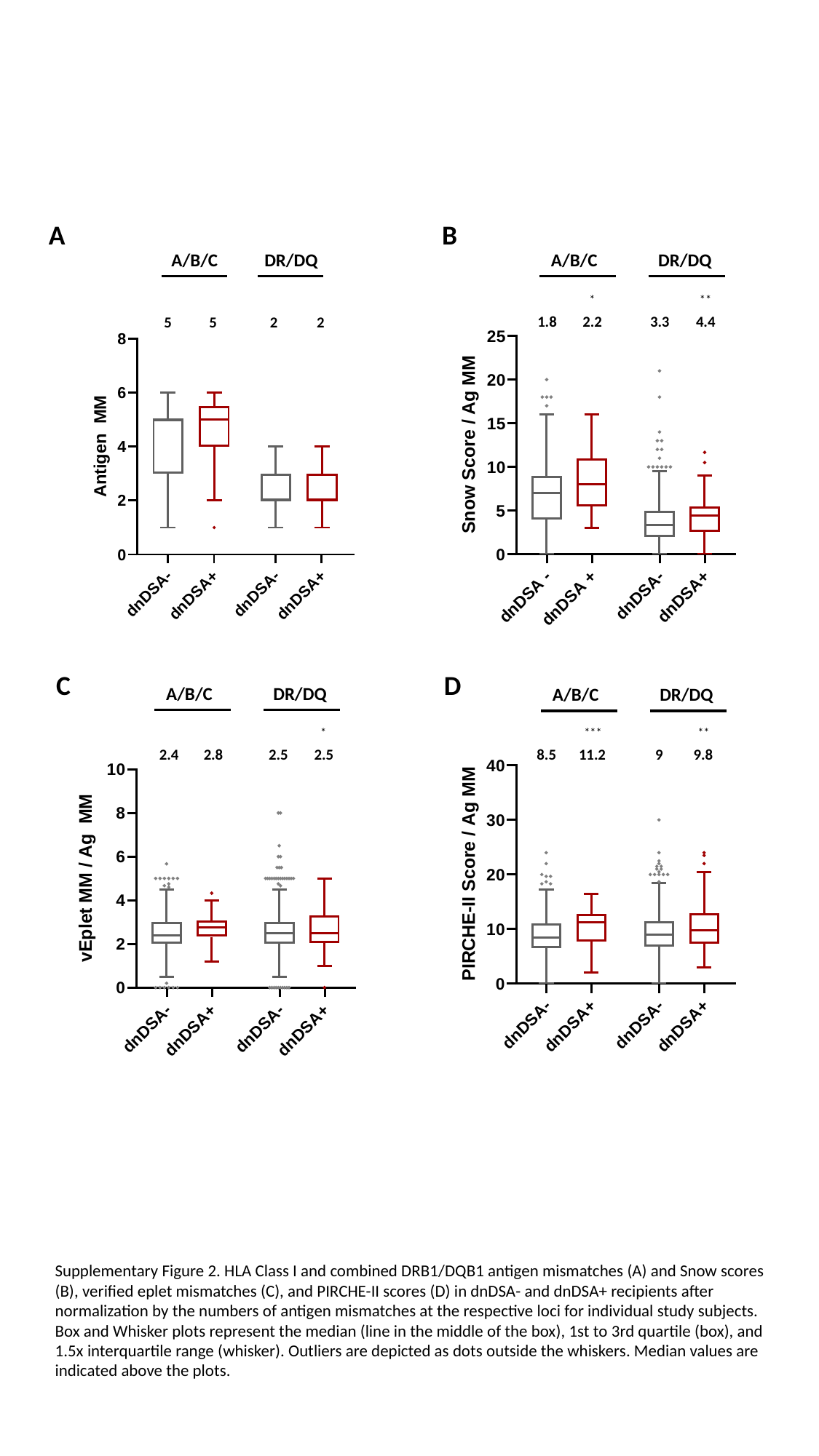

A
B
A/B/C
DR/DQ
A/B/C
DR/DQ
*
**
1.8
2.2
3.3
4.4
5
5
2
2
C
D
A/B/C
DR/DQ
A/B/C
DR/DQ
*
***
**
2.4
2.8
2.5
2.5
8.5
11.2
9
9.8
Supplementary Figure 2. HLA Class I and combined DRB1/DQB1 antigen mismatches (A) and Snow scores (B), verified eplet mismatches (C), and PIRCHE-II scores (D) in dnDSA- and dnDSA+ recipients after normalization by the numbers of antigen mismatches at the respective loci for individual study subjects. Box and Whisker plots represent the median (line in the middle of the box), 1st to 3rd quartile (box), and 1.5x interquartile range (whisker). Outliers are depicted as dots outside the whiskers. Median values are indicated above the plots.

## Slide 6
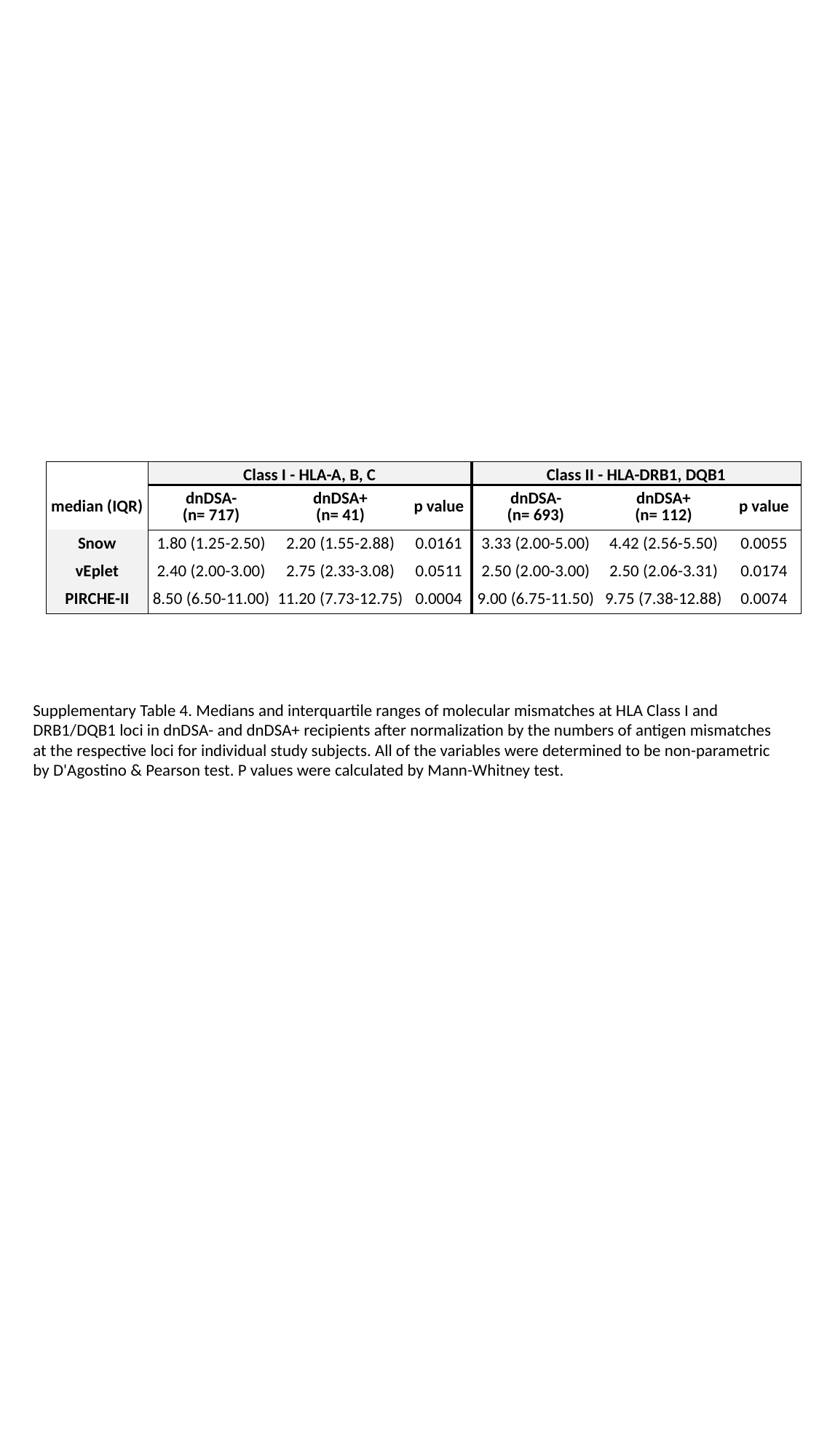

| | Class I - HLA-A, B, C | | | Class II - HLA-DRB1, DQB1 | | |
| --- | --- | --- | --- | --- | --- | --- |
| median (IQR) | dnDSA- (n= 717) | dnDSA+ (n= 41) | p value | dnDSA- (n= 693) | dnDSA+ (n= 112) | p value |
| Snow | 1.80 (1.25-2.50) | 2.20 (1.55-2.88) | 0.0161 | 3.33 (2.00-5.00) | 4.42 (2.56-5.50) | 0.0055 |
| vEplet | 2.40 (2.00-3.00) | 2.75 (2.33-3.08) | 0.0511 | 2.50 (2.00-3.00) | 2.50 (2.06-3.31) | 0.0174 |
| PIRCHE-II | 8.50 (6.50-11.00) | 11.20 (7.73-12.75) | 0.0004 | 9.00 (6.75-11.50) | 9.75 (7.38-12.88) | 0.0074 |
Supplementary Table 4. Medians and interquartile ranges of molecular mismatches at HLA Class I and DRB1/DQB1 loci in dnDSA- and dnDSA+ recipients after normalization by the numbers of antigen mismatches at the respective loci for individual study subjects. All of the variables were determined to be non-parametric by D'Agostino & Pearson test. P values were calculated by Mann-Whitney test.

## Slide 7
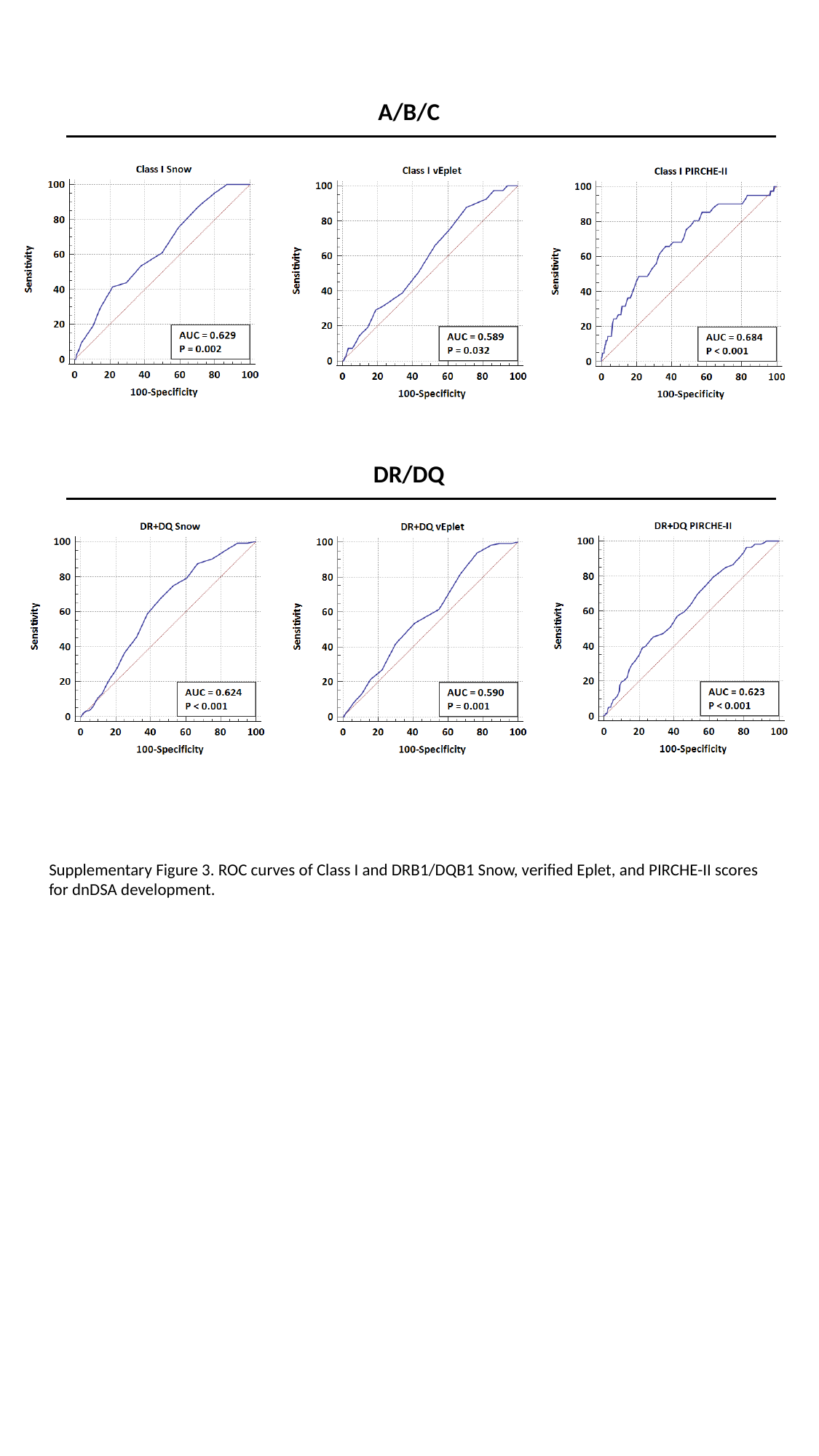

A/B/C
DR/DQ
Supplementary Figure 3. ROC curves of Class I and DRB1/DQB1 Snow, verified Eplet, and PIRCHE-II scores for dnDSA development.

## Slide 8
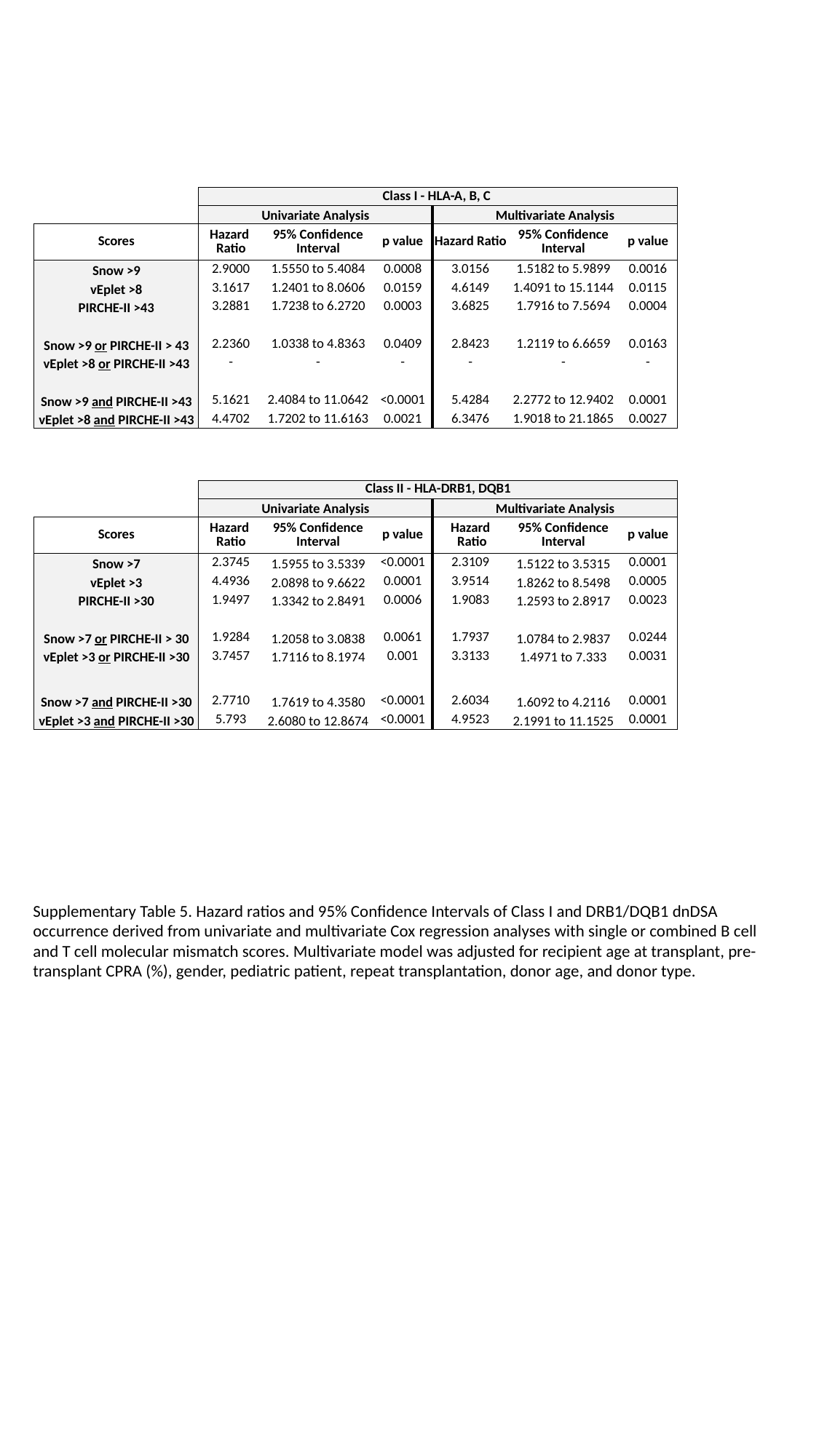

| | Class I - HLA-A, B, C | | | | | |
| --- | --- | --- | --- | --- | --- | --- |
| | Univariate Analysis | | | Multivariate Analysis | | |
| Scores | Hazard Ratio | 95% Confidence Interval | p value | Hazard Ratio | 95% Confidence Interval | p value |
| Snow >9 | 2.9000 | 1.5550 to 5.4084 | 0.0008 | 3.0156 | 1.5182 to 5.9899 | 0.0016 |
| vEplet >8 | 3.1617 | 1.2401 to 8.0606 | 0.0159 | 4.6149 | 1.4091 to 15.1144 | 0.0115 |
| PIRCHE-II >43 | 3.2881 | 1.7238 to 6.2720 | 0.0003 | 3.6825 | 1.7916 to 7.5694 | 0.0004 |
| | | | | | | |
| Snow >9 or PIRCHE-II > 43 | 2.2360 | 1.0338 to 4.8363 | 0.0409 | 2.8423 | 1.2119 to 6.6659 | 0.0163 |
| vEplet >8 or PIRCHE-II >43 | - | - | - | - | - | - |
| | | | | | | |
| Snow >9 and PIRCHE-II >43 | 5.1621 | 2.4084 to 11.0642 | <0.0001 | 5.4284 | 2.2772 to 12.9402 | 0.0001 |
| vEplet >8 and PIRCHE-II >43 | 4.4702 | 1.7202 to 11.6163 | 0.0021 | 6.3476 | 1.9018 to 21.1865 | 0.0027 |
| | | | | | | |
| | | | | | | |
| | | | | | | |
| | Class II - HLA-DRB1, DQB1 | | | | | |
| | Univariate Analysis | | | Multivariate Analysis | | |
| Scores | Hazard Ratio | 95% Confidence Interval | p value | Hazard Ratio | 95% Confidence Interval | p value |
| Snow >7 | 2.3745 | 1.5955 to 3.5339 | <0.0001 | 2.3109 | 1.5122 to 3.5315 | 0.0001 |
| vEplet >3 | 4.4936 | 2.0898 to 9.6622 | 0.0001 | 3.9514 | 1.8262 to 8.5498 | 0.0005 |
| PIRCHE-II >30 | 1.9497 | 1.3342 to 2.8491 | 0.0006 | 1.9083 | 1.2593 to 2.8917 | 0.0023 |
| | | | | | | |
| Snow >7 or PIRCHE-II > 30 | 1.9284 | 1.2058 to 3.0838 | 0.0061 | 1.7937 | 1.0784 to 2.9837 | 0.0244 |
| vEplet >3 or PIRCHE-II >30 | 3.7457 | 1.7116 to 8.1974 | 0.001 | 3.3133 | 1.4971 to 7.333 | 0.0031 |
| | | | | | | |
| Snow >7 and PIRCHE-II >30 | 2.7710 | 1.7619 to 4.3580 | <0.0001 | 2.6034 | 1.6092 to 4.2116 | 0.0001 |
| vEplet >3 and PIRCHE-II >30 | 5.793 | 2.6080 to 12.8674 | <0.0001 | 4.9523 | 2.1991 to 11.1525 | 0.0001 |
Supplementary Table 5. Hazard ratios and 95% Confidence Intervals of Class I and DRB1/DQB1 dnDSA occurrence derived from univariate and multivariate Cox regression analyses with single or combined B cell and T cell molecular mismatch scores. Multivariate model was adjusted for recipient age at transplant, pre-transplant CPRA (%), gender, pediatric patient, repeat transplantation, donor age, and donor type.

## Slide 9
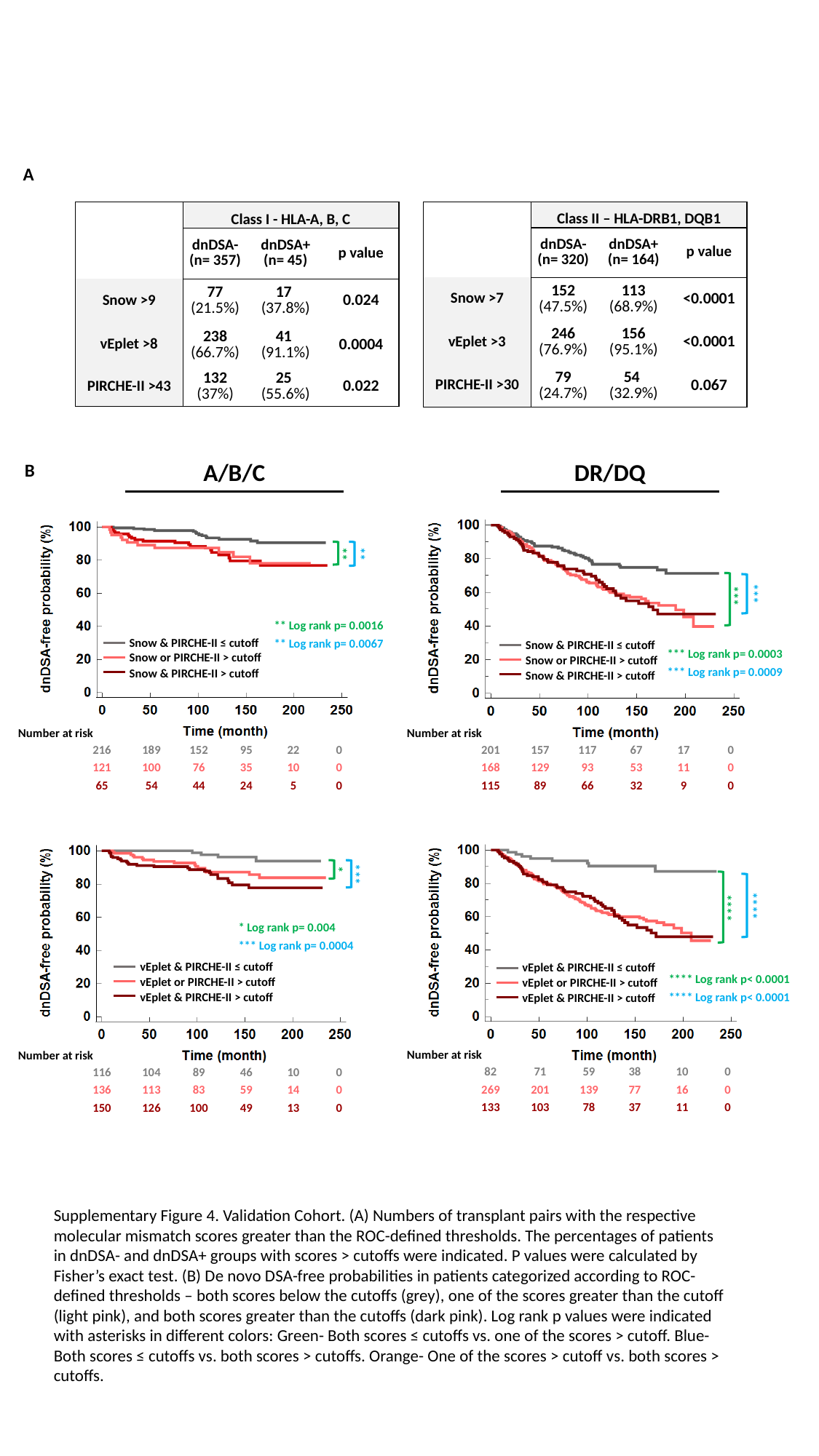

A
| | Class I - HLA-A, B, C | | |
| --- | --- | --- | --- |
| | dnDSA- (n= 357) | dnDSA+ (n= 45) | p value |
| Snow >9 | 77 (21.5%) | 17 (37.8%) | 0.024 |
| vEplet >8 | 238 (66.7%) | 41 (91.1%) | 0.0004 |
| PIRCHE-II >43 | 132 (37%) | 25 (55.6%) | 0.022 |
| | Class II – HLA-DRB1, DQB1 | | |
| --- | --- | --- | --- |
| | dnDSA- (n= 320) | dnDSA+ (n= 164) | p value |
| Snow >7 | 152 (47.5%) | 113 (68.9%) | <0.0001 |
| vEplet >3 | 246 (76.9%) | 156 (95.1%) | <0.0001 |
| PIRCHE-II >30 | 79 (24.7%) | 54 (32.9%) | 0.067 |
A/B/C
DR/DQ
B
**
**
***
***
** Log rank p= 0.0016
** Log rank p= 0.0067
Snow & PIRCHE-II ≤ cutoff
Snow & PIRCHE-II ≤ cutoff
*** Log rank p= 0.0003
*** Log rank p= 0.0009
Snow or PIRCHE-II > cutoff
Snow or PIRCHE-II > cutoff
Snow & PIRCHE-II > cutoff
Snow & PIRCHE-II > cutoff
Number at risk
Number at risk
216
189
152
95
22
0
201
157
117
67
17
0
121
100
76
35
10
0
168
129
93
53
11
0
65
54
44
24
5
0
115
89
66
32
9
0
*
***
****
****
* Log rank p= 0.004
*** Log rank p= 0.0004
vEplet & PIRCHE-II ≤ cutoff
vEplet & PIRCHE-II ≤ cutoff
**** Log rank p< 0.0001
**** Log rank p< 0.0001
vEplet or PIRCHE-II > cutoff
vEplet or PIRCHE-II > cutoff
vEplet & PIRCHE-II > cutoff
vEplet & PIRCHE-II > cutoff
Number at risk
Number at risk
82
71
59
38
10
0
116
104
89
46
10
0
269
201
139
77
16
0
136
113
83
59
14
0
133
103
78
37
11
0
150
126
100
49
13
0
Supplementary Figure 4. Validation Cohort. (A) Numbers of transplant pairs with the respective molecular mismatch scores greater than the ROC-defined thresholds. The percentages of patients in dnDSA- and dnDSA+ groups with scores > cutoffs were indicated. P values were calculated by Fisher’s exact test. (B) De novo DSA-free probabilities in patients categorized according to ROC-defined thresholds – both scores below the cutoffs (grey), one of the scores greater than the cutoff (light pink), and both scores greater than the cutoffs (dark pink). Log rank p values were indicated with asterisks in different colors: Green- Both scores ≤ cutoffs vs. one of the scores > cutoff. Blue- Both scores ≤ cutoffs vs. both scores > cutoffs. Orange- One of the scores > cutoff vs. both scores > cutoffs.
